# Gaussian process test for high-throughput sequencing time series: application to experimental evolution

**DOI:** 10.1093/bioinformatics/btv014

**Published:** 2015-01-21

**Authors:** Hande Topa, Ágnes Jónás, Robert Kofler, Carolin Kosiol, Antti Honkela

**Affiliations:** ^1^Helsinki Institute for Information Technology (HIIT), Department of Information and Computer Science, Aalto University, Espoo, Finland, ^2^Institut für Populationsgenetik, Vetmeduni Vienna, 1210 Wien, Austria, ^3^Vienna Graduate School of Population Genetics, Wien, Austria and ^4^Helsinki Institute for Information Technology (HIIT), Department of Computer Science, University of Helsinki, Helsinki, Finland

## Abstract

**Motivation:** Recent advances in high-throughput sequencing (HTS) have made it possible to monitor genomes in great detail. New experiments not only use HTS to measure genomic features at one time point but also monitor them changing over time with the aim of identifying significant changes in their abundance. In population genetics, for example, allele frequencies are monitored over time to detect significant frequency changes that indicate selection pressures. Previous attempts at analyzing data from HTS experiments have been limited as they could not simultaneously include data at intermediate time points, replicate experiments and sources of uncertainty specific to HTS such as sequencing depth.

**Results:** We present the beta-binomial Gaussian process model for ranking features with significant non-random variation in abundance over time. The features are assumed to represent proportions, such as proportion of an alternative allele in a population. We use the beta-binomial model to capture the uncertainty arising from finite sequencing depth and combine it with a Gaussian process model over the time series. In simulations that mimic the features of experimental evolution data, the proposed method clearly outperforms classical testing in average precision of finding selected alleles. We also present simulations exploring different experimental design choices and results on real data from Drosophila experimental evolution experiment in temperature adaptation.

**Availability and implementation:** R software implementing the test is available at https://github.com/handetopa/BBGP.

**Contact:** hande.topa@aalto.fi, agnes.jonas@vetmeduni.ac.at, carolin.kosiol@vetmeduni.ac.at, antti.honkela@hiit.fi

**Supplementary information: **Supplementary data are available at *Bioinformatics* online.

## 1 Introduction

Most biological processes are dynamic and analysis of time series data is necessary to understand them. Recent advances in high-throughput sequencing (HTS) technologies have provided new experimental approaches to collect genome-wide time series. For example, experimental evolution now uses a new evolve and re-sequencing (ER) approach to understand which genes are targeted by selection and how (Burke and Long, 2012; Kawecki *et al.*, 2012). Such experiments enable phenotypic divergence to be forced in response to changes in only few environmental conditions in the laboratory while other conditions are kept constant. The evolved populations are then subjected to HTS.

Experimental evolution in microorganisms has focused on the fate new mutations. For example, in *Escherichia coli* (Barrick *et al.*, 2009) and *Saccharomyces cerevisae* (Lang *et al.*, 2013) new mutations were studied. In contrast, ER experiments with sexually reproducing multicellular organisms address selection on standing variation and allele frequency changes (AFCs) in small populations where drift plays an important role. For example, for *Drosophila melanogaster* (*Dmel*), several phenotypic traits, such as accelerated development (Burke *et al.*, 2010), body size variation (Turner *et al.*, 2011), hypoxia-tolerance (Zhou *et al.*, 2011) and temperature adaptation (Orozco-terWengel *et al.*, 2012) have been investigated. Motivated by these experimental studies, we believe that experimental evolution combined with HTS supplies a good basis for studying AFC through time series molecular data.

To perform allele frequency comparisons, pairwise statistical tests between base and evolved populations were typically carried out. Burke *et al.* (2010) combined Fisher’s exact tests with a sliding-window approach to identify genomic regions that show allele frequency differences between populations selected for accelerated development and controls without direct selection. Turner *et al.* (2011) developed a pairwise summary statistic, called ‘diff-Stat’ to estimate the observed distribution of allele frequency differences and compared this to the expected distribution without selection. Orozco-terWengel *et al.* (2012) identify single nucleotide polymorphisms (SNPs) with a consistent AFC among replicates by performing a Cochran-Mantel-Haenszel test (CMH) (Agresti, 2002). The latter is an extension of the Fisher’s exact test to multiple replicates. All aforementioned statistical methods are based on pairwise comparisons between the base and evolved populations and they do not take full advantage of the time series data now available. Bollback *et al.* (2008) developed a method to analyze time series data based on population genetic models and estimated the effective population size *N_e_* of a bacteriophage from a single locus. Illingworth *et al.* (2012) derived a model for time series data from large populations of microorganisms ($N_{e}\approx 10^{8}$) where drift can be ignored and the population allele frequencies evolve ‘quasi-deterministically’. Here, we propose an alternative Gaussian process (GP) based approach to study AFCs over the entire time series experiment genome-wide for small populations ($N_{e}\approx 10^{2}-10^{3}$).

GP is a non-parametric statistical model that is extremely well suited for modelling HTS time series data, which usually have relatively few time points that may be irregularly sampled. Recently, there have been some works applying GP models with parameters describing the process of evolution (e.g. Jones and Moriarty, 2013 account for phylogenetic relationships, Palacios and Minin, 2013 for effective population size). GPs have also recently been applied to gene expression time series by a number of authors (Äijö *et al.*, 2013; Cooke *et al.*, 2011; Gao *et al.*, 2008; Hensman *et al.*, 2013; Honkela *et al.*, 2010; Kalaitzis and Lawrence, 2011; Kirk and Stumpf, 2009; Liu *et al.*, 2010; Liu and Niranjan, 2012; Stegle *et al.*, 2010; Titsias *et al.*, 2012; Yuan, 2006). In differential analysis, GPs have been applied to detect differences in gene expression time series in a two-sample setting by Stegle *et al.* (2010) and for detecting significant changes by Kalaitzis and Lawrence (2011). Although these methods provide a sensible basis for detecting the changing alleles, they fail to properly take into account all aspects of the available HTS data, such as differences in sequencing depth between different alleles and time points. These differences can have a huge impact in the reliability of different measured allele frequencies and taking them into account is vital for achieving good accuracy with the available short time series.

## 2 Methods

To identify the candidate alleles which evolve under selection, we model the allele frequencies by GP regression. We fit time-dependent and time-independent GP models and rank the alleles according to their corresponding Bayes factors (BFs), i.e. the ratio of the marginal likelihoods under the different models.

GPs provide a convenient approach for modelling short time series. However, when applying them to a large number of short parallel time series as in many genomic applications, naive application leads to overfitting or underfitting in some examples. Although these problems are rare, the bad examples can easily dominate the ranking. We overcome these challenges by excluding non-sensical parameter values, for example using a good variance model that can be incorporated into the GP models.

### 2.1 Data and preprocessing

In the following, we use the term SNP for the markers and alleles under study, but the methods can be applied to any features whose abundance can be quantified in a similar manner. We consider SNPs that are bi-allelic for a specific position of the genome in a population. Multi-allelic SNPs, however, exist but are rare and likely to be sequencing errors (Burke *et al.*, 2010). Multi-allelic cases can be treated by simply ignoring the least frequent allele or transformed to bi-allelic site by summing up the frequencies of the most infrequent alleles. Here, we assumed that only two of the alleles from (A, T, C, G) can be observed at each SNP position. After determining the abundances of these two specific alleles, we model the time dependency of the rising allele’s frequency over several generations. We will refer to generations as time points for simplicity.

We denote the replicate index of each observation by *r_j_* and the time point by *t_j_*, *j* = 1,…, *J*, with *J* denoting the total number of observations. For each of these points, we assume HTS reads have been aligned to a reference genome with *y_ij_* reads with a specific allele at SNP position *i*. We use *n_ij_* to denote the total sequencing depth at the position.

### 2.2 Mean and variance inference: beta-binomial model

We model *y_ij_* as a draw from a binomial distribution with parameters *n_ij_* and *p_ij_*:

(1) $$y_{ij}|n_{ij},p_{ij}∼\text{Bin}(n_{ij},p_{ij}),$$

where *p_ij_* denotes the frequency of the specific allele in the population. We set a uniform Beta(1,1) prior on *p_ij_*:

(2) $$p_{ij}|\alpha ,\beta ∼\text{Beta}(\alpha ,\beta ),$$

where *α* = 1, *β* = 1.

Since beta prior is conjugate to the binomial likelihood, the posterior distribution will also be a beta distribution:

(3) $$p_{ij}|y_{ij},n_{ij},\alpha ,\beta ∼\text{Beta}(\alpha_{ij}^{*},\beta_{ij}^{*}),$$

where

$$\alpha_{ij}^{*}=\alpha +y_{ij},$$

$$\beta_{ij}^{*}=\beta +n_{ij}-y_{ij}.$$

Then, the posterior mean and variance of *p_ij_* can be calculated as follows:

(4) $$\text{E}(p_{ij}|y_{ij},n_{ij},\alpha ,\beta )=\frac{\alpha_{ij}^{*}}{\alpha_{ij}^{*}+\beta_{ij}^{*}}=\frac{\alpha +y_{ij}}{\alpha +\beta +n_{ij}}$$

(5) Var(pij|yij,nij,α,β)=αij*βij*(αij*+βij*)2(αij*+βij*+1)=(α+yij)(β+nij−yij)(α+β+nij)2(α+β+nij+1).

**Fig. 1.**
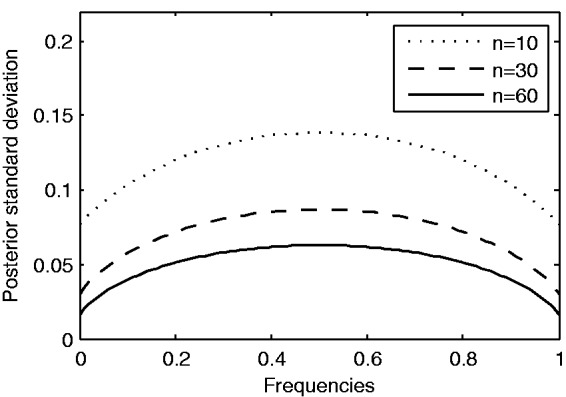
Posterior standard deviations of the allele frequencies with sequencing depths 10, 30 and 60

The inferred posterior means and posterior variances are used to fit the GP models as described in the following sections. As the results will show, this step is very important for incorporating the available uncertainty information into the GP models by taking into account different sequencing depths. For example, beta-binomial model assigns larger variances to the alleles with lower sequencing depths (Fig. 1). Moreover, the Beta(1,1) prior on *p_ij_* leads to a symmetry in the posterior mean and variance. Therefore, the result of our method is not affected whichever allele is chosen from the alternative alleles.

### 2.3 GP regression

A GP is a collection of random variables, any finite number of which has a joint Gaussian distribution. We write

(6) f(t)∼GP(m(t),K(t,t′))

to denote that *f*(*t*) follows a GP with mean function m(t)=E[f(t)] and covariance function K(t,t′)=E[(f(t)−m(t))(f(t′)−m(t′))]. We let $y={\left(y_{i}\right)}_{i=1}^{N}$ be a vector of the noisy observations measured at points $t={\left(t_{i}\right)}_{i=1}^{N}$ satisfying

(7) $$y_{i}=f(t_{i})+\epsilon ,$$

where *ϵ* is Gaussian observation noise with zero mean and a diagonal covariance matrix $\Sigma_{\epsilon }$. To simplify the algebra, we assume the mean function *m*(*t*) = 0 and subtract the mean of **y**.

Gaussian processes allow marginalizing the latent function to obtain a marginal likelihood. The covariance function *K* and the noise covariance $\Sigma_{\epsilon }$ depend on hyperparameters *θ* that can be estimated by maximizing the log marginal likelihood:

(8) log⁡(p(y|t,θ))=−12yT[K(t,t)+Σϵ]−1y−12log⁡|K(t,t)+Σϵ|−N2log⁡(2π),

where K(t,t) denotes the covariance matrix constructed by evaluating the covariance function at points **t**. It is also possible to compute the posterior mean and covariance at non-sampled time points $t_{*}$, given the noisy observations **y** at sampled time points **t**. This is often useful for visualization purposes. We obtain (Rasmussen and Williams, 2006):

(9) $$f_{*}|y∼\text{N}(m_{*},\Sigma_{*}),$$

where

$$\begin{array}{l} m_{*}=\text{E}[f_{*}|y]=K(t_{*},t){\left[K(t,t)+\Sigma_{\epsilon }\right]}^{-1}y,\\ \Sigma_{*}=K(t_{*},t_{*})-K(t_{*},t){\left[K(t,t)+\Sigma_{\epsilon }\right]}^{-1}K(t,t_{*}). \end{array}$$

**Fig. 2.**
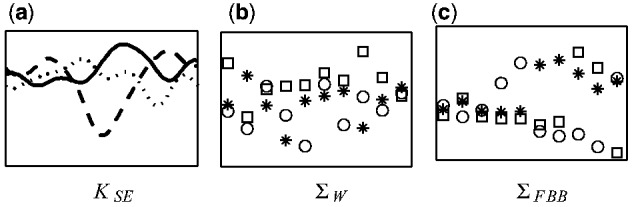
Example realizations from GPs and noise processes with different covariance structures

In our GP models, we use the squared exponential covariance matrix to model the underlying smooth function. The squared exponential covariance

(10) $$K_{\text{SE}}(t,t\prime )=\sigma_{f}^{2}e^{\left(-\frac{{\left(t-t\prime \right)}^{2}}{2l^{2}}\right)}$$

has two parameters: the length scale, *l*, and the signal variance, $\sigma_{f}^{2}$. Length scale specifies the distance beyond which any two inputs become uncorrelated. A small length scale means that the function fluctuates very quickly, whereas a large length scale means that the function behaves like a constant function. Three example realizations generated with squared exponential covariance matrix can be seen in Figure 2a.

In the standard GP model, the observation noise is assumed to be white: the noise at different time points is independent and identically distributed. The corresponding covariance matrix

(11) $$\Sigma_{\epsilon }=\Sigma_{W}=\sigma_{n}^{2}I$$

is an identity matrix multiplied by the noise variance parameter, $\sigma_{n}^{2}$. Three example realizations generated with white noise covariance matrix can be seen in Figure 2b.

### 2.4 Beta-binomial Gaussian process

The beta-binomial Gaussian process (BBGP) method combines beta-binomial model with the GP model in the sense that the posterior means and posterior variances of the frequencies, which are inferred by beta-binomial model, are used to fit the GP model by means of an additional noise covariance matrix which we call fixed beta-binomial (FBB) covariance matrix.

Returning to Section 2.2, let us denote the posterior mean and variance of *p_ij_* by *m_ij_* and $s_{ij}^{2}$, respectively. That is,

(12) $$m_{ij}=\text{E}(p_{ij}|y_{ij},n_{ij},\alpha ,\beta )$$

(13) $$s_{ij}^{2}=\text{Var}(p_{ij}|y_{ij},n_{ij},\alpha ,\beta ).$$

To fit the BBPG model, we assume

(14) $$m_{ij}=f_{i}(t_{j})+\mu_{m_{i}}+\epsilon ,$$

where $f_{i}(t)∼GP(0,K_{\text{SE}}(t,t\prime ))$ and $\epsilon ∼N(0,\Sigma_{W}+\Sigma_{\text{FBB}})$. The mean $\mu_{m_{i}}$ is eliminated by subtracting the mean from *m_ij_*. Because of Σ_FBB_ this is an approximation that may fail if *n_ij_* vary significantly, but it speeds up inference significantly. The additional covariance

(15) $$\Sigma_{\text{FBB}}=\text{diag}(s_{ij}^{2})$$

is a diagonal FBB covariance matrix which is used to include known variance information for each observation in the GP model. The elements of Σ_FBB_ are determined by the posterior variances which are inferred from beta-binomial model in Section 2.2. Three example realizations generated with FBB covariance matrix can be seen in Figure 2c, where larger variance values were inferred for the later time points.

### 2.5 BBGP-based test

**Fig. 3.**
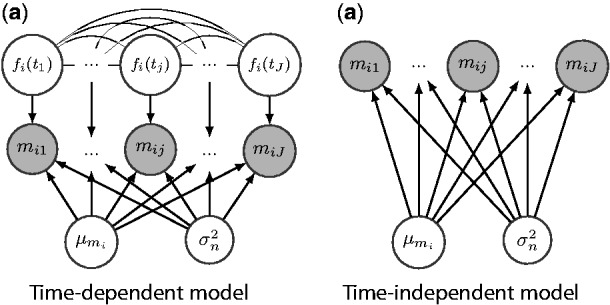
Graphical models for the (**a**) time-dependent and (**b**) time-independent BBGP models

**Fig. 4.**
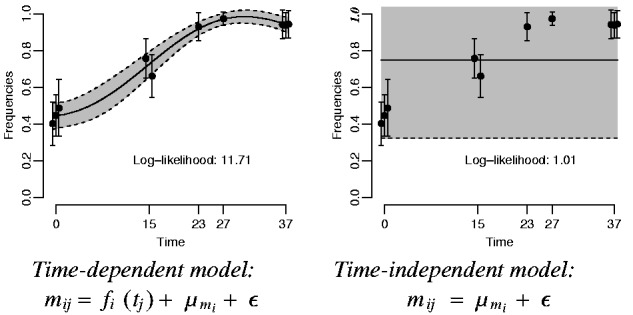
BBGP fits for the time-dependent and time-independent models for an example SNP taken from the real dataset (Orozco-terWengel *et al*., 2012). Confidence regions are shown for ± 2 standard deviation. Similarly, error bars indicate ± 2 standard deviation (from FBB) interval. Replicates at the same time points are shifted by 0.5 for better visualization. Maximum likelihood estimates of the parameters: ${\hat{\theta }}_{1}=\{\hat{\ell }=15.53,\;\hat{\sigma_{f}^{2}}=0.05,\;\hat{\sigma_{n}^{2}}=3.6\times 10^{-8}\}$; ${\hat{\theta }}_{2}=\{\hat{\sigma_{n}^{2}}=0.05\}$

We fit the ‘time-dependent’ BBGP model of Equation (14) and a ‘time-independent’ model without the GP term $f_{i}(t_{j})$ for each SNP *i*. As can be seen from the graphical models in Figure 3, ‘time-independent’ model assumes that the observations are randomly generated around a constant mean with no temporal dependency, whereas ‘time-dependent’ model captures the dependency between the observations by the function $f_{i}(t)$, which follows a GP with the squared exponential covariance function. Thereby the parameters of the squared exponential covariance [*K*_SE_, Equation (10)] in the time-dependent model and the white noise covariance [Σ*_W_*, Equation (11)] in both models are fitted by maximizing the marginal likelihood. The FBB covariance [Σ_FBB_, Equation (15)] does not contain any free hyperparameters. If the model is actually time independent, the length scale in the squared exponential covariance is estimated to be very large, which makes the maximum likelihood of the time-dependent model equivalent to that of time-independent model. Figure 4 shows an example of the time-dependent (left) and time-independent (right) BBGP models.

We maximize the log marginal likelihood functions for the models by scaled conjugate gradient method using the ‘gptk’ R package by Kalaitzis and Lawrence (2011). We use a grid search over the parameter space and initialize the parameters to the grid value with highest likelihood. We also set a lower bound equal to the shortest spacing between observations for the length scale parameter to avoid overfitting.

We compute the BF for SNP *i* as (Kalaitzis and Lawrence, 2011; Stegle *et al.*, 2010)

(16) $${\text{BF}}_{i}=\frac{p(m_{i}|{\hat{\theta }}_{1},\text{“time-dependent model ”})}{p(m_{i}|{\hat{\theta }}_{2},\text{“time-independent model}”)},$$

where ${\hat{\theta }}_{1}$ and ${\hat{\theta }}_{2}$ contain the maximum likelihood estimates of the hyperparameters in the corresponding BBGP models. BFs indicate the degree of the models to be ‘time dependent’ rather than ‘time independent’.

### 2.6 CMH test

We compare BBPG against the CMH test, which was used by Orozco-terWengel *et al.* (2012) to identify alleles with consistent AFC across replicates. The CMH test has been proven to be the best-performing test statistic applied on HTS evolutionary data so far (Kofler and Schlötterer, 2014). Therefore, we take it as the basis of comparison with BBGP.

CMH allows to test whether the joint odds ratio of replicated (r=1,…,R) allele counts in a 2×2×R contingency table (Table 1) is significantly different from one. Significant deviation from one implies dependence of allele counts between two time points that is consistent among replicates. The CMH tests pairwise observations of the two alternative allele counts $y_{ij}^{\left(1\right)}$ and $y_{ij}^{\left(2\right)}$. In our bi-allelic case $y_{ij}^{\left(1\right)}=y_{ij}$ and $y_{ij}^{\left(2\right)}=n_{ij}-y_{ij}$. To compare the counts for all replicates r=1,…,R at the base (B) and the end (E) time points for each SNP position *i*, we denote $B_{r}=\{j|t_{j}=B,r_{j}=r\}$ and $E_{r}=\{j|t_{j}=E,r_{j}=r\}$. The CMH test statistic [see Agresti (2002) and Supplementary Text Section S1] compares the cell counts in Table 1 to their null expected values and it follows a chi-squared distribution with one degree of freedom $X_{\left(df=1\right)}^{2}$. We performed CMH tests on the simulated and real data for each SNP position independently, using the implementation of the software PoPoolation2 (Kofler *et al.*, 2011).

**Table 1.** 2 × 2 contingency table of allele counts for the *r*-th replicate

	Base generation (B)	End generation (E)	∑
**SNP** *i* **allele 1**	$y_{iB_{r}}^{\left(1\right)}$	$y_{iE_{r}}^{\left(1\right)}$	$y_{i._{r}}^{\left(1\right)}$
**SNP** *i* **allele 2**	$y_{iB_{r}}^{\left(2\right)}$	$y_{iE_{r}}^{\left(2\right)}$	$y_{i._{r}}^{\left(2\right)}$
∑	$n_{iB_{r}}$	$n_{iE_{r}}$	$n_{i._{r}}$

### 2.7 Simulations

To evaluate the performances of the BBGP and the CMH tests, we simulated data that mimic the dynamics of evolving *Dmel* populations at the genomic level. For this aim, we first simulated three sets of genome-scale data to evaluate the overall performances of the methods under the experimental design which is close to the natural settings. Additionally, we also carried out smaller size simulations on one chromosome arm to investigate the further influences of different parameter settings on the methods.

#### 2.7.1 Whole-genome simulations

We carried out forward Wright-Fisher simulations of genome-wide allele frequency trajectories of populations using the MimicrEE simulation tool (Kofler and Schlötterer, 2014). The initial haplotypes were taken from Kofler and Schlötterer (2014), and they capture the natural variation of *Dmel* population. By sampling from the initial set, we established *r* = 5 replicated base populations using *H* = 200 founder haplotypes and let each of them evolve for *g* = 60 generations at a constant census size of *N* = 1000. We used the spatially varying recombination rate defined for *Dmel* by Fiston-Lavier *et al.* (2010). Low recombining regions were excluded from the simulations because of the elevated false-positive rate in these regions (Kofler and Schlötterer, 2014). We followed the evolution of the total number of 19 39 941 autosomal SNPs among which 100 were selected with selection coefficient of *s* = 0.1 and semi-dominance (*h* = 0.5). Furthermore, we required the selected SNPs to have a starting frequency in the range [0.12,0.8], not to lose the minor allele in the course of time due to drift. We recorded the nucleotide counts for every second generation and performed Poisson sampling with *λ* = 45 (overall mean coverage in Orozco-terWengel *et al.*, 2012) on the count data to produce coverage information (see Supplementary Text Section S2). We repeated the whole simulation experiment three times, each time using a different set of selected SNPs.

#### 2.7.2 Single-chromosome-arm simulations

For experimental design, additional simulations were carried out on a single chromosome arm (∼16 Mb) with 25 selected SNPs to assess the performance under various parameter combinations, such as population size (*N*), number of founder haplotypes (*H*), selection coefficient (*s*), level of dominance (*h*), number of generations (*g*) and number of replicates (*r*). We defined a basic set up with parameter space close to that of the whole-genome simulations, i.e. N=1000,H=200,r=5,g=60,s=0.1,h=0.5, and investigated the effect on the performance when only one parameter is perturbed from its basic value.

### 2.8 Evaluation metrics

The methods were evaluated based on precision, recall and average precision (AP) (Manning *et al.*, 2008). Precision and recall are commonly used metrics to measure the fraction of relevant items that are retrieved when comparing ranking based methods. Precision and recall are defined as

(17) $$\text{pre}(k)=\frac{\text{number of selected SNPs in }k\text{ top SNPs}}{k},$$

(18) $$\text{rec}(k)=\frac{\text{number of selected SNPs in }k\text{ top SNPs}}{\text{number of selected SNPs}}.$$

The curve obtained by plotting the precision at every position in the ranked sequence of items as a function of recall is called the precision-recall curve. The area under the curve can be summarized using AP (Manning *et al.*, 2008), which is defined as the average of pre(*k*) after every returned selected SNP:

(19) $$\text{ave}P=\frac{\sum_{k=1}^{N}(\text{pre}(k)1_{\text{sel}}(k))}{\text{number of selected SNPs}},$$

where *N* is the total number of SNPs and
(20)
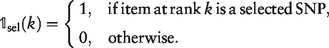



## 3 Results

### 3.1 Simulated whole-genome data

We applied the BBGP and CMH on the genome-wide simulated data with different numbers of time points (i.e. generations) and replicates. To evaluate the effect of the number of time points used, we tested the method using subsets of different sizes of the nine time points {0,6,14,22,28,38,44,50,60} (see Supplementary Text Section S3 for details). We performed BBGP separately for each of the sampling schemes while CMH can only use two time points (first and last). All simulated SNPs were scored using BFs for the BBGP, and *P*-values for the CMH test (e.g. see Supplementary Fig. S1 for a graphical visualization of the scores).

To investigate the effect of the number of replicates (*r*), we chose up to five replicates at each sampled time point. We first performed CMH tests with all possible *r*-replicate combinations. We then applied BBGP only to the best performing replicate combinations of each size according to AP in the CMH evaluations. This strategy ensures a fair comparison between the methods as BBGP is always evaluated against the best CMH results. We also compared BBGP to the standard GP of Kalaitzis and Lawrence (2011) that does not use the FBB model variances using the same replicate combinations as BBGP with 6 time points.

**Fig. 5.**
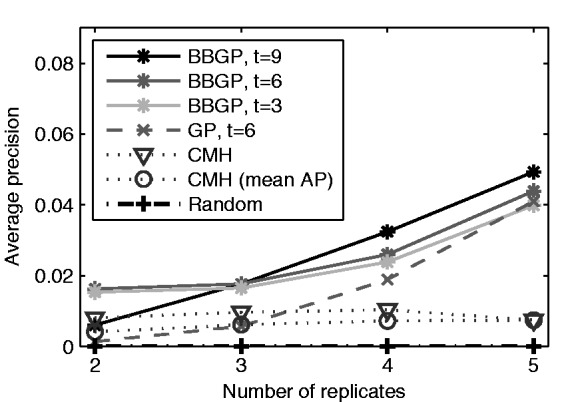
APs for CMH, BBGP and standard GP with different number of replicates (*r*) and time points (*t*) in the whole-genome simulation. The best-performing *r*-replicate combination in the CMH test has been used in GP and BBGP, and the mean AP for CMH has been computed by taking the mean of the APs over all *r*-replicate combinations for *r* = 2,3,4,5. The corresponding precision–recall curves are shown in Supplementary Figures S2 and S3. Additionally, average AP for random ranking (line of no discrimination) is shown by a constant line at the true fraction of selected SNPs in the simulated data, i.e. $100/1,939,941\approx 5*10^{-5}$

As shown in Figure 5 (see also Supplementary Figs. S2 and S3), BBGP achieves a higher AP than the standard GP and the CMH. Somewhat surprisingly, CMH seems to benefit very little from more replicates while the performance of the GP methods improves noticeably. The CMH is sensitive to the specific replicates included, as including the fifth replicate in the optimal sequence actually leads to worse performance than four replicates (Supplementary Fig. S3c and d). We did not observe similar behaviour with the GP methods. On average over all possible *r*-replicate combinations, adding more replicates helps the CMH as well (mean AP in Fig. 5). The performance of the standard GP approaches that of BBGP as the number of replicates increases, which is consistent with the view that the stronger prior information from sequencing depth is most important when the data are otherwise scarce, as is often the case in real experiments. In contrast to more replicates, adding more time points improved BBGP's performance very little (Fig. 5).

We also investigated whether the two methods identify different types of selected SNPs. We calculated AFC for each SNP based on the average difference between the base and end populations across replicates. The CMH is sensitive to large AFCs, while the candidates detected by the BBGP have a much more uniform distribution of AFCs (Supplementary Fig. S5). In general, we would expect a uniform distribution of AFCs, as very large AFCs are only possible for SNPs with low starting frequency giving them the potential to rapidly increase. BBGP is much more accurate than CMH in all AFC classes as demonstrated by the performance breakdown in Supplementary Figure S6.

Furthermore, we performed a generalized CMH test (gCMH) that can be applied to more than two time points but requires a weighting scheme (Supplementary Text Section S1.1). As there is no straightforward way to find weights that accurately reflects natural selection, we used mid-ranks assigned to time points. The performance of the gCMH drops rapidly with increasing the number of time points and replicates (Supplementary Fig. S7), which might be due to a poor weighting scheme.

The performance of the methods can vary noticeably between different experiments depending on their difficulty. For example, there is a 10-fold difference in AP between Experiment 1 and Experiment 3 for both methods (Supplementary Fig. S4, see also Kofler and Schlötterer (2014) for the CMH), but the BBGP-based test consistently outperforms the CMH test.

The running time needed to analyze 1000 SNPs in a 4-replicate 6-time point setting is ≈30 min on a desktop running Ubuntu 12.04 with Intel(R) Xeon(R) CPU E3-1230 V2 at 3.30 GHz. 

### 3.2 Influence of parameter choice

For the purpose of experimental design, we investigated further parameter settings on the single chromosome arm of 2L.

#### 3.2.1 Population size and number of founder haplotypes

**Fig. 6.**
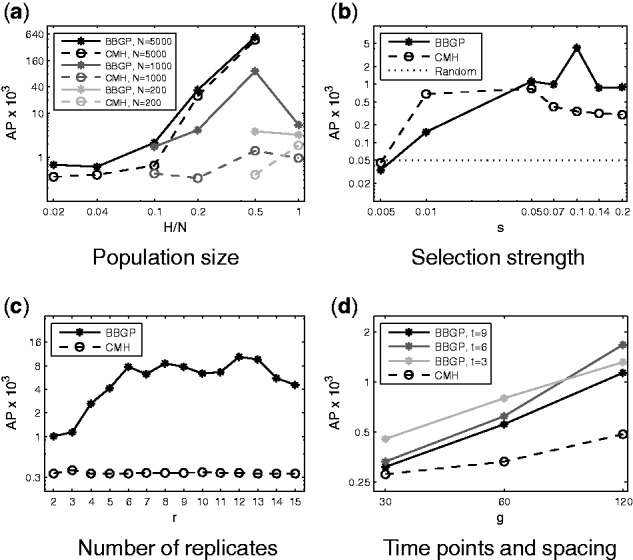
APs for different experimental designs. AP for random ranking is shown in (**b**) by a constant line at the true value of the ratio of selected SNPs in the simulated data, i.e. $25/496,611\approx 5*10^{-5}$. Log scale was used on both axes for (**a**), (b), (**d**) and on the *y*-axis for (**c**). Other parameters are as in the basic setup in Section 2.7

In finite populations, genetic drift has a large impact on shaping the population allele frequencies. We studied the effect of census populations size (*N*) and the number of founder haplotypes (*H*). *H* can be thought as the number of different individuals (isofemale lines) in the base population. The populations were established by randomly choosing *N* individuals with replacement out of the *H* founders. The simulation results show that AP increases with increasing *N* (Fig. 6a). This has also been observed by Kofler and Schlötterer (2014) for the CMH test. The AP is the highest with the ratio of H/N=0.5 in all cases (Fig. 6a, Supplementary Figs. S8–S10) and the BBGP consistently outperforms the CMH test. Kofler and Schlötterer (2014) reported that the true-positive rate for CMH test increases with *H* but the increment levels off with H/N=0.5 for *N* = 1000. Baldwin-Brown *et al.* (2014) detected a constant increase in the power to localize a candidate SNP; however, they used a different method and investigated different parameter settings not comparable to ours. We hypothesize that as more low-frequency variants are present in the population with H/N>0.5, the selected SNPs with multiple linked backgrounds are competing with each other, resulting in an AP drop.

#### 3.2.2 Selection strength and level of dominance

We investigated the performance using various selection coefficients (*s*) and fixed semidominance (*h* = 0.5). For moderate and strong selection (*s* > 0.01), the BBGP outperforms the CMH test (Fig. 6b, Supplementary Fig. S11). The BBGP reaches the highest precision at *s* = 0.1, whereas the CMH test is the most precise at *s* = 0.05 which is consistent with Kofler and Schlötterer (2014). For strong selection (*s* = 0.2) the precision drops for both methods. The performance decay is presumably due to interference between selected sites, known as the Hill–Robertson effect, i.e. linkage between sites under selection will reduce the overall effectiveness of selection in finite populations (Hill and Robertson, 1966). Also, we hypothesize that long-range associations become more apparent as the strength of selection increases (Supplementary Fig. S12) resulting in larger blocks rising in frequency together, which was also observed by Tobler *et al.* (2014).

For weak selection (s≤0.01), it becomes hard to distinguish between selection and drift in small populations. Thus, for low *s*, both methods perform rather poorly and the CMH has a slightly higher AP in these cases. However, for a more ideal parameter choice of N=5000,H=2500 and a long runtime of the experiment (*g* = 120), the BBGP gains a large performance improvement over the CMH test for *s* = 0.01 (see Supplementary Figs. S13 and S14) even in the difficult scenario of weak selection.

We also simulated evolving populations using different levels of dominance (*h*). The following relative fitness values were used on genotypes *AA*, *Aa* and *aa*: $w_{AA}=1+s,\;w_{Aa}=1+hs$, *w_aa_* = 1, where *s* = 0.1. As *h* varies, we observed different behaviour of the methods. The AP of the CMH test increases as we are moving from complete recessivity (*h* = 0, recessive phenotype is selected) to complete dominance (*h* = 1, dominant phenotype is selected) (Supplementary Figs. S15 and S16). Selection on completely recessive allele results in a gradual initial change in AF with more rapid change in later generations and eventual fixation. In contrast, the change in AF of a completely dominant allele is initially rapid but never reaches fixation as the recessive allele is shielded from natural selection in the heterozygote. When the fitness of the heterozygote is intermediate between the two homozygotes (additivity, *h* = 0.5) the allele frequency trajectory is the combination of the aforementioned ones, i.e. rapid initial change and quick fixation. BBGP reaches the highest AP with the additive scenario and relatively high AP in the recessive case (Supplementary Fig. S15). When the dominant phenotype is selected (h∼1) and the unfavoured allele stays present in the population at low frequency, it is likely to result in an inconsistent behaviour of replicates, which lower the power of the BBGP.

#### 3.2.3 Number of replicates

In addition to the whole-genome experiments with a maximum of five replicates, we simulated up to *r* = 15 replicates for the single chromosome arm. We observed a constant increase in performance for the BBGP up to *r* = 6 (Fig. 6c, Supplementary Fig. S17). The AP kept increasing up to *r* = 12 but rather in a fluctuating manner and then dropped with adding even more replicates. Consistently with the whole-genome simulations, we did not observe a large performance improvement with increasing the number of replicates for the CMH test.

#### 3.2.4 Length of the experiment and spacing of the samples

We also examined the performance with increasing the length of the experiments up to *g* = 120 generations. For longer experiments, more recombination events can happen, which uncouples linked sites letting them evolve independently. The AP rises rapidly for longer experiments (Fig. 6d, Supplementary Fig. S18). Thereby the performance gain is noticeably higher for the BBGP. We also investigated the spacing of the sampled time points (t∈{3,6,9}) for the BBGP and observed similar pattern that of the whole-genome simulations, i.e. an intermediate number of sample time points is sufficient as shape of selected trajectories is simple.

### 3.3 Real data application

Orozco-terWengel *et al.* (2012) applied ER methods on *Dmel* populations adapting to elevated temperature regime. They established base populations from isofemale lines collected in Portugal. The populations were propagated at a constant size of 1000 for 37 generations under fluctuating temperature regime (12h at 18°C and 12h at 28°C). DNA pools of 500 females (Pool-Seq) were sequenced at the following time points: three replicates at the base generation 0 (B); two replicates at generation 15, an additional replicate at generation 23 and 27; three replicates at the end generation 37 (E).

**Fig. 7.**
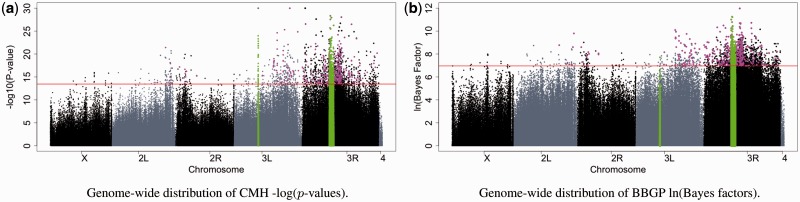
Manhattan plots of genome-wide SNP-values. (**a**) $-\log_{10}(P\text{-values})$ for the CMH test B-E comparison. *P*-values below 1e−30 were clipped to 1e−30 on the plot. (**b**) ln (BFs) for the BBGP. Only those SNPs are indicated for which we calculated both the *P*-values and the BFs (we did not infer BFs for tri-allelic SNPs). A 1 Mb region was excluded from the analysis on 3R as a low-frequency haplotype spreads during the experiment. Previously, the chorion gene cluster on 3L was also excluded as this region has extremely high coverage (Orozco-terWengel *et al.*, 2012). Regions that were excluded from the analysis are shown in green. The red horizontal line indicates the top 2000 candidate cutoff. The common candidates among the top 2000 are highlighted in magenta. Figure 7(b) shows how well the beta-binomial variance control can handle high coverage problem of the excluded region on 3L

CMH tests were performed on a SNP-wise basis to identify significant AFCs between the B and E populations [see Orozco-terWengel *et al.* (2012) and Supplementary Text Section S4]. We applied the BBGP method on 12 57 117 SNPs and compared the results with that of the B-E comparison of the CMH test. The overlap between the top 2000 candidate SNPs of the CMH and the BBGP was rather small (609 SNPs). However, the peaks of both methods covered the same regions (Fig. 7).

The difference between the methods is illustrated with some example allele trajectories in Supplementary Figures S23–S25. BBGP emphasizes between-replicate consistency and sometimes picks candidate SNPs that start already at high frequency and go rapidly to fixation. On the other hand, CMH test assigns high ranks to SNPs with large frequency change and fails to detect between-replicate consistency if the fold change is otherwise low.

Using a gene set enrichment analysis (see Supplementary Text Section S5), we also found that the top ranked significantly enriched Gene Ontology categories were similar for both tests (Supplementary Tables S3 and S4, Supplementary Fig. S19). Furthermore, Figure 7 shows how well the posterior beta-binomial variance inference can handle false signals resulting from uneven coverage. While the CMH test is misled by strong signal coming from high coverage of the chorion cluster with high copy number variation, the BBGP test does not falsely indicate signatures of selection (Fig. 7, green region on 3L).

Although *Dmel* generally has rather small levels of linkage, linkage disequilibrium (LD) might have built up during the course of the experiment. In fact, LD had a major effect on the number of candidate SNPs identified by the CMH as well as the BBGP test. As the flanking SNPs showed signs of hitchhiking, the observed AFC of the flanking SNPs were also significant (see also Manhattan plot for the simulated SNPs Supplementary Fig. S1), and this made it difficult to narrow down functionally important regions for thermoadaptation.

## 4 Discussion

Our results in detecting SNPs that are evolving under selection using a GP model clearly demonstrate the importance of careful modelling of the measurement uncertainty through a good noise model, in our case using the beta-binomial model of sequencing data. Especially when data are scarce, the BBGP approach leads to much higher accuracy than standard maximum likelihood estimation of noise variances. Incorporating the non-Gaussian likelihood directly to the GP would also be possible, but it would lead to computationally more demanding inference.

In terms of experimental design, the most effective way to improve performance is to use a larger population (*N*) and a larger number of founder haplotypes (*H*). As expected, alleles under moderate to strong selection (*s* = 0.05–0.1) are easier to detect than alleles changing under weak selection (s≤0.01). However, for very strong selection (s≥0.2), it is again hard to detect the causal SNPs. In a real experiment, the strength of selection might also not be known and often cannot be changed for the trait of interest. Adding more replicates can also help improve performance up to some point. Compared with the CMH test, the BBGP is clearly superior in utilizing additional replicates. We suspect this is because CMH assumes all replicates should have similar odds ratios between the two time points and this is not sufficiently satisfied by the noisy data. Longer experiments can help significantly (Supplementary Fig. S13), but the benefit of adding more intermediate time points seems smaller. This may be because the shape of selected trajectories is a simple sigmoid, and adding more points provides limited help in estimating them. The presented GP-based test is sensitive to SNPs with a consistent time-varying profile. A statistically more accurate model could be derived by assuming each replicate to follow an independent GP, but this would require different kind of constraints to differentiate between selection and drift, which may be difficult to formulate for multiple interacting SNPs. Exploring hierarchical GP models to capture the correct dependence structure is an interesting avenue of future research.

In a whole-genome experiment, LD between nearby markers and interactions between nearby selected SNPs are important confounders in identifying the selected markers. Especially for moderate-sized populations, the interactions can be problematic, leading to very large segments in the genome raising together in frequency (Supplementary Fig. S12). The issue does not appear when simulating only a single selected SNP (Supplementary Fig. S20), which strongly suggests it is caused by the interactions. The issue can be most effectively mitigated by using larger populations (Supplementary Fig. S21c and d). An artificially high recombination rate (Supplementary Fig. S21a and b) could also break the interactions. Working with larger fixed window sizes might not improve the performance as a substantial number of hitchhikers can still be found hundreds of kilobases from the selected SNPs (See Supplementary Fig. S22: The removal of nearby hitchhikers did not improve the AP noticeably). It is possible to extend the GP models for joint analysis of multiple SNPs, and this is clearly an important avenue of future research. This is potentially a further advantage of the GP, because it is much more difficult to similarly extend the frequentist tests.

## 5 Conclusion

In this article, we developed a new test that is based on combining GP models with a beta-binomial model of sequencing data, and compared it with the CMH test that allows the pairwise comparison of base and evolved populations across several replicates.

Our results demonstrate that GP models are well suited for analyzing quantitative genomic time series data because they can effectively utilize the available data, making good use of additional time points and replicates unhindered by uneven sampling and consistently show performance superior to the CMH test.

The GP framework is very flexible, which enables extensions utilizing for example LD over nearby alleles. As GP models can easily incorporate additional information on the data, we envisage that further promising combinations of the GP approach with evolutionary models will emerge. 

## Supplementary Material

Supplementary Data

## References

[btv014-B1] Agresti A. (2002) Categorical Data Analysis*.* Wiley, New York.

[btv014-B2] Äijö T. (2013) Sorad: a systems biology approach to predict and modulate dynamic signaling pathway response from phosphoproteome time-course measurements. Bioinformatics*,* 29, 1283–1291. 23505293 10.1093/bioinformatics/btt130

[btv014-B3] Baldwin-Brown J.G. (2014) The power to detect quantitative trait loci using resequenced, experimentally evolved populations of diploid, sexual organisms. Mol. Biol. Evol.*,* 31, 1040–1055. 24441104 10.1093/molbev/msu048 PMC3969567

[btv014-B4] Barrick J.E. (2009) Genome evolution and adaptation in a long-term experiment with *Escherichia coli*. Nature*,* 461, 1243–1247. 19838166 10.1038/nature08480

[btv014-B5] Bollback J.P. (2008) Estimation of 2Nes from temporal allele frequency data. Genetics*,* 179, 497–502. 18493066 10.1534/genetics.107.085019 PMC2390626

[btv014-B6] Burke M. (2010) Genome-wide analysis of a long-term evolution experiment with Drosophila. Nature*,* 467, 587–590. 20844486 10.1038/nature09352

[btv014-B7] Burke M.K. Long A (2012) What paths do advantageous alleles take during short-term evolutionary change? *Mol**.* Ecol.*,* 21, 4913–416. 10.1111/j.1365-294x.2012.05745.x 23227489

[btv014-B8] Cooke E.J. (2011) Bayesian hierarchical clustering for microarray time series data with replicates and outlier measurements. BMC Bioinformatics*,* 12, 399. 21995452 10.1186/1471-2105-12-399 PMC3228548

[btv014-B9] Fiston-Lavier A.S. (2010) *Drosophila melanogaster* recombination rate calculator. Gene*,* 463, 18–20. 20452408 10.1016/j.gene.2010.04.015

[btv014-B10] Gao P. (2008) Gaussian process modelling of latent chemical species: applications to inferring transcription factor activities. Bioinformatics*,* 24, i70–i75. 18689843 10.1093/bioinformatics/btn278

[btv014-B11] Hensman J. (2013) Hierarchical Bayesian modelling of gene expression time series across irregularly sampled replicates and clusters. BMC Bioinformatics*,* 14, 252. 23962281 10.1186/1471-2105-14-252 PMC3766667

[btv014-B12] Hill W.G. Robertson A (1966) The effect of linkage on limits to artificial selection. Genet. Res.*,* 8, 269–294. 5980116

[btv014-B13] Honkela A. (2010) Model-based method for transcription factor target identification with limited data. Proc. Natl Acad. Sci. USA*,* 107, 7793–7798. 20385836 10.1073/pnas.0914285107 PMC2867914

[btv014-B14] Illingworth C.J.R. (2012) Quantifying selection acting on a complex trait using allele frequency time series data. Mol. Biol. Evol.*,* 29, 1187–1197. 22114362 10.1093/molbev/msr289 PMC3731369

[btv014-B15] Jones N.S. Moriarty J (2013) Evolutionary inference for function-valued traits: Gaussian process regression on phylogenies. J. R. Soc. Interface*,* 10, 20120616. 23135249 10.1098/rsif.2012.0616 PMC3565790

[btv014-B16] Kalaitzis A.A. Lawrence N.D (2011) A simple approach to ranking differentially expressed gene expression time courses through Gaussian process regression. BMC Bioinformatics*,* 12, 180. 21599902 10.1186/1471-2105-12-180 PMC3116489

[btv014-B17] Kawecki T.J. (2012) Experimental evolution. Trends. Ecol. Evol.*,* 27, 547–560. 22819306 10.1016/j.tree.2012.06.001

[btv014-B18] Kirk P.D.W. Stumpf M.P.H (2009) Gaussian process regression bootstrapping: exploring the effects of uncertainty in time course data. Bioinformatics*,* 25, 1300–1306. 19289448 10.1093/bioinformatics/btp139 PMC2677737

[btv014-B19] Kofler R. Schlötterer C (2014) A guide for the design of evolve and resequencing studies. Mol. Biol. Evol.*,* 31, 474–483. 24214537 10.1093/molbev/mst221 PMC3907048

[btv014-B20] Kofler R. (2011) PoPoolation2: identifying differentiation between populations using sequencing of pooled DNA samples (Pool-Seq). Bioinformatics*,* 27, 3435–3436. 22025480 10.1093/bioinformatics/btr589 PMC3232374

[btv014-B21] Lang G.I. (2013) Pervasive genetic hitchhiking and clonal interference in forty evolving yeast populations. Nature*,* 500, 571–574. 23873039 10.1038/nature12344 PMC3758440

[btv014-B22] Liu Q. (2010) Estimating replicate time shifts using Gaussian process regression. Bioinformatics*,* 26, 770–776. 20147305 10.1093/bioinformatics/btq022 PMC2832819

[btv014-B23] Liu W. Niranjan M (2012) Gaussian process modelling for bicoid mRNA regulation in spatio-temporal Bicoid profile. Bioinformatics*,* 28, 366–372. 22130592 10.1093/bioinformatics/btr658

[btv014-B24] Manning C.D. (2008) Introduction to Information Retrieval*.* Cambridge University Press, Cambridge, England.

[btv014-B25] Orozco-terWengel P. (2012) Adaptation of Drosophila to a novel laboratory environment reveals temporally heterogeneous trajectories of selected alleles. Mol. Ecol.*,* 21, 4931–4941. 22726122 10.1111/j.1365-294X.2012.05673.x PMC3533796

[btv014-B26] Palacios J.A. Minin V.N (2013) Gaussian process-based Bayesian nonparametric inference of population size trajectories from gene genealogies. Biometrics*,* 69, 8–18. 23409705 10.1111/biom.12003

[btv014-B27] Rasmussen C.E. Williams C.K.I. (2006) Gaussian Processes for Machine Learning*.* The MIT Press, Cambridge, MA.

[btv014-B28] Stegle O. (2010) A robust Bayesian two-sample test for detecting intervals of differential gene expression in microarray time series. J. Comput. Biol.*,* 17, 355–367. 20377450 10.1089/cmb.2009.0175 PMC3198888

[btv014-B29] Titsias M.K. (2012) Identifying targets of multiple co-regulating transcription factors from expression time-series by Bayesian model comparison. BMC Syst. Biol.*,* 6, 53. 22647244 10.1186/1752-0509-6-53 PMC3527261

[btv014-B30] Tobler R. (2014) Massive habitat-specific genomic response in *D. melanogaster* populations during experimental evolution in hot and cold environments*. *Mol. Biol. Evol.*,* 31, 364–375. 24150039 10.1093/molbev/mst205 PMC3907058

[btv014-B31] Turner T. (2011) Population-based resequencing of experimentally evolved populations reveals the genetic basis of body size variation in *Drosophila melanogaster*. PLoS Genet.*,* 7, e1001336. 21437274 10.1371/journal.pgen.1001336 PMC3060078

[btv014-B32] Yuan M (2006) Flexible temporal expression profile modelling using the Gaussian process. Comput. Statist. Data Anal., 51, 1754–1764.

[btv014-B33] Zhou D. (2011) Experimental selection of hypoxia-tolerant *Drosophila melanogaster*. Proc. Natl Acad. Sci. USA*,* 7, 2349–2354. 21262834 10.1073/pnas.1010643108 PMC3038716

