# Supplementary material for: Gaussian process test for high-throughput sequencing time series: application to experimental evolution
Source: Bioinformatics. 2015 Jan 21;31(11):1762–70. doi: 10.1093/bioinformatics/btv014 (PMC4443671; doi:10.1093/bioinformatics/btv014)
Supplement: Supplementary Data [file supp_31_11_1762__index.html]

Gaussian process test for high-throughput sequencing time series: application to experimental evolution — Gaussian process test for high-throughput sequencing time series: application to experimental evolution — Gaussian process test for high-throughput sequencing time series: application to experimental evolution — Supplementary Data 

# Gaussian process test for high-throughput sequencing time series: application to experimental evolution

## Supplementary Data

files

**Files in this Data Supplement:**

- Supplementary Data - pdf file
