# Supplementary material for: Gaussian process test for high-throughput sequencing time series: application to experimental evolution
Source: Bioinformatics. 2015 Jan 21;31(11):1762–70. doi: 10.1093/bioinformatics/btv014 (PMC4443671; doi:10.1093/bioinformatics/btv014)
Supplement: Supplementary Data [file supp_btv014_minorRevision_BBGP_supplement.pdf]

Supplementary Text

Gaussian process test for high-throughput sequencing time series: application to experimental evolution

Hande Topa, Ágnes Jónás, Robert Kofler, Carolin Kosiol, Antti Honkela

1 Cochran-Mantel-Haenszel Test

SNPs with consistent change in allele frequency were identified with Cochran-Mantel-Haenszel test (CMH) by Orozco-Ter Wengel *et al.* (2012). The CMH test is an extension of testing equivalence of proportions (implies that the odds ratio is 1) in a 2×2 contingency table to replicated tables sampled from the same underlying population. The estimate for the joint odds ratio in the replicated $2 \times 2 \times R$ tables ($r = 1, \ldots, R$, Main Text, Table 1) is tested for difference from 1.

We follow the definition of the CMH by Agresti (2002). Allele counts for the different replicates ($y^{(1)}_{iB_r}$, Main Text, Table 1) are assumed to be independent. Under the null hypothesis, they follow a hypergeometric distribution with mean and variance:

$$E(y^{(1)}_{iB_r}) = \frac{y^{(1)}_{i.r} n_{iB_r}}{n_{i.r}}$$

$$V(y^{(1)}_{iB_r}) = \frac{y^{(1)}_{i.r} y^{(2)}_{i.r} n_{iB_r} n_{iE_r}}{n^2_{i.r}(n_{i.r} - 1)}.$$

The test statistic compares $\sum_r y^{(1)}_{iB_r}$ to its null expected value by combining information from R partial tables:

$$CMH = \frac{\left[\sum_r \left(y^{(1)}_{iB_r} - E(y^{(1)}_{iB_r})\right)\right]^2}{\sum_r Var\left(y^{(1)}_{iB_r}\right)}.$$

This statistic approximately follows a chi-square distribution with one degree of freedom $\chi^2_{(df=1)}$. Under the null hypothesis, we assume independence of the start (B) and end (E) time points of the experiment for each replicate. Thus, the odds ratio for each replicate is approximately one. When the odds ratio in each partial table is significantly different from one (dependence) we expect the nominator in the test statistic to be large in absolute value.

1.1 Generalized Cochran-Mantel-Haenszel Test (gCMH)

The CMH tests for associations between pairwise allele counts and it is not able to handle time serial data. However, it can be generalized for $K \times L \times R$ contingency tables (Kuritz

et al., 1988) where the null hypothesis of no partial association between the row ($i = 1, \dots, K$) dimensions and column ($j = 1, \dots, L$) dimensions for all replicates ($r = 1, \dots, R$) is tested. Similarly to the CMH test, under the null hypothesis the cell counts do not deviate from their expected value under random association. The alternative hypothesis can vary depending on whether the row and column variables are measured in the nominal or ordinal scale. In the HTS allele frequency data, the row variable (allele A and B) is nominal, whereas the column variable (allele counts at different time points) is measured on the ordinal scale. We test the alternative hypothesis that mean allele frequency across several time points differ between alleles. Mean allele frequencies are formed by assigning (column) scores to time points and the difference between the weighted mean scores across rows are tested (see e.g. Kuritz *et al.* (1988) for details). There is no straightforward way to find a proper weighting scheme of the time points, which accurately reflects the action of natural selection. We used the `R` implementation of the generalized CMH test in `vcdExtra` (Friendly, 2014) package where mid-ranks can be assigned to column scores (`cscores="midrank"`). Using these marginal ranks obtained form each table, the test statistic is equivalent to an extension of Kruskal-Wallis analysis of variance test on ranks.

To our knowledge, the gCMH test has not been used to analyse HTS allele frequency data. We used it on our simulated whole-genome data set to see if performance improvement can be achieved when time serial information is incorporated to the CMH test. We performed gCMH with increasing number of replicates using $t = 3, 6, 9$ time points (Fig. S7). With less time points ($t = 3$, Fig. S7(a)) the gCMH does better but the performance drops with increasing the number of time points. Generally, we also see a precision decline as the number of replicates rises.

2 Simulations

We carried out whole-genome forward Wright-Fisher simulations of allele frequency (AF) trajectories of evolving populations with MimicrEE (Kofler and Schlötterer, 2014). The founder population was generated using 8000 simulated haploid genomes from Kofler and Schlötterer (2014). Out of the 8000 genomes, 200 were sampled to establish a diploid base population of 1000 individuals (sampled out of the 200 with replacement). The base population contains only autosomal SNPs. Low recombining regions ($< 1cM/Mb$) were also excluded from the simulations (for more information see Kofler and Schlötterer, 2014). We randomly placed 100 selected SNPs in the base population with selection coefficient of $s = 0.1$ and semi-dominance ($h = 0.5$). The selected SNPs have a starting allele frequency in the range $[0.12, 0.8]$. We applied this restriction on the staring AF to increase the probability of fixation of the selected allele. According to population genetics theory, the probability of fixation is $P_{fix} = (1 - e^{-2N_e sp})/(1 - e^{-2N_e s})$ (Kimura, 1962), where N_e is the effective population size, s is the selection coefficient and p is the starting allele frequency. Taking the base population of 1000 homozygote individuals and the set of selected SNPs, we followed the simulation protocol outlined at https://code.google.com/p/mimicree/wiki/ManualMimicrEESummary for 5 replicates independently. As described in Kofler and Schlötterer (2014), we aimed to reproduce the sampling properties of Pool-Seq using Poisson sampling with $\lambda = 45$ (using the script poisson-3fold-sample.py available at http://mimicree.googlecode.com). Briefly, we considered coverage differences between samples, coverage fluctuations due to GC-bias and stochastic sampling heterogeneity.

3 Performance tests on simulated data

We measured the performance of the BBGP and the CMH test using whole-genome simulated data with various number of time points and replicates. To evaluate the effect of the number of time points used, the following sampling schemes were carried out. We started with nine time points $\{0, 6, 14, 22, 28, 38, 44, 50, 60\}$ and then removed the midpoint of the shortest interval until the desired number of time points was achieved. In the case of a tie, we kept the time point which is closest to the real sequenced time points in Orozco-Ter Wengel *et al.* (2012). Following this rule, we applied BBGP on the following sets of generations:

- 3 time points: 0, 38, 60,
- 4 time points: 0, 14, 38, 60,
- 5 time points: 0, 14, 28, 38, 60,
- 6 time points: 0, 14, 28, 38, 50, 60,
- 7 time points: 0, 14, 22, 28, 38, 50, 60,
- 8 time points: 0, 6, 14, 22, 28, 38, 50, 60,
- 9 time points: 0, 6, 14, 22, 28, 38, 44, 50, 60.

For the CMH test, however, we always performed a base-end (generation 60) comparison, because the CMH is a pairwise statistic. The genome-wide test statistic values are shown in Figure S1 for the BBGP (6 time points) and the CMH for 5 replicates as an example. The effects of different numbers of replicates on the performance of the proposed methods are shown in Figure S3 using precision recall curves along with average precisions. We carried out 3 independent runs of simulations with different sets of selected SNPs but keeping the parameters unchanged (Fig. S4). Finally, we compared with a performance break down according to Allele Frequency Change (AFC) the BBGP to CMH test in different AFC classes (Fig. S5 and Fig. S6).

3.1 Tests of parameter choice for experimental design

We investigated different choices of parameters for experimental design. As whole-genome simulations are computationally very demanding, we decided to simulate only a single chromosome arm (2L) with 25 selected SNPs using various parameter settings. This reduces the running times significantly, but the length of the genome segment ($\sim 16Mb$) and the number of selected SNPs used are still realistic proxy to the performance on the whole-genome. We report performance results for different population size - number of founder haplotypes ($\frac{H}{N}$ combinations (Fig. S8 - S10), for various selection coefficients s (Fig. S11-S14), levels of dominance h (Fig. S15, S16), increasing number of replicates r (Fig. S17) and the choice of time points at different intermediate generations g (Fig S18).

4 Real Data Application

We applied the BBGP on HTS data of experimentally evolved *D. melanogaster* populations (Orozco-Ter Wengel *et al.*, 2012). We compared our proposed method to the CMH results coming from the B-E comparison, downloaded from Dryad database (http://datadryad.org) under the accession: doi: 10.5061/dryad.60k68. We used the synchronized pileup files (BF37.sync)

which contains a total number of 1,547,837 SNPs. The CMH test was only performed on 1,547,764 SNPs that met certain quality criteria regarding the minor allele count and the maximum coverage (for more information on SNP calling please consult Orozco-Ter Wengel *et al.*, 2012). We also excluded the tri-allelic SNPs in our analyses, which resulted in 1,257,117 SNPs in total.

Example allele trajectories can be seen in Fig. S23 - S25 for the candidate SNPs detected by either CMH or BBGP, or both. The figures show that between-replicate consistency is hugely important for BBGP while the candidates which do not have this consistency can be falsely picked up by CMH. On the other hand, CMH fails to detect highly consistent data if the fold change is too small, which is in line with our observation in Fig. S5(d).

5 Gene Set Enrichment

We used gene set enrichment to test for significantly enriched functional categories according to the Gene Ontology (GO) database (Ashburner *et al.*, 2000). Orozco-Ter Wengel *et al.* (2012) used Gowinda (Kofler and Schlötterer, 2012) to test significance of overrepresentation of candidate SNPs in each GO category. Gowinda uses permutation tests to eliminate potential sources of bias caused by difference of gene length and genes that overlap (explained below). We tested the top 2000 candidate SNPs for both the CMH and the BBGP methods, respectively. FDR correction was applied on the inferred p-values to account for multiple testing. Using Gowinda, we did only find one significantly enriched category ($p < 0.05$) for the BBGP and no significant categories for the CMH test (see Tables S1 and S2).

In addition to taking an arbitrary threshold of the top 2000 SNPs, we also considered the full distributions of p-values for the CMH and the distribution of Bayes factors for the BBGP based tests. For each GO category we compared distribution of all SNP-values (p-values for the CMH and Bayes factors for the GP) in that GO gene set to the distribution outside that gene set using a one-tailed Mann-Whitney U test (MWU) as applied by Segrè *et al.* (2010). Similar to Gowinda, we used permutations to account for biases such as gene length and other confounding effects (see below). We also conserve the gene order during the randomization as functionally similar genes are often clustered nearby on a chromosome. Using the MWU tests, we found significant GO category enrichments for both methods (Fig. S19). Moreover, the top ranked candidate categories were similar in both cases (see Table S3, S4).

5.1 Gene Set Enrichment with Gowinda

Gowinda counts the number of genes (set of candidate genes) that contain candidate SNPs. Assuming that SNPs are in complete linkage within the same gene, it randomly samples SNPs from the pool of all SNPs until the number of corresponding genes is equal to the cardinality of the set of candidate genes. This step is repeated several times and from the resulting random set of genes, an empirical null distribution of candidate gene abundance is calculated for each gene set. The significance level of enrichment for each gene set is inferred by counting the randomly drawn cases, in which there were more candidate genes present than in the original candidate gene set. Gowinda requires the following input files: annotation file containing the annotation of species of interest; gene set file of the associated genes (e.g. Gene Ontology (GO) association file); list of SNP-value pairs as the output of our analysis; list of candidate SNPs, which is a subset of all SNP-value pairs that we define as candidates according to some predetermined condition. We used the following inputs: the annotation file of

Drosophila melanogaster version 5.40 downloaded from Flybase (http://flybase.org/); the GO association file was obtained from R Bioconductor GO.db package version 2.9.0 (accessed at 05/03/2013). We took the top 2000 candidate SNPs for both methods as candidate SNPs and run Gowinda with the following parameters: `--simulations 10000000 --gene-definition updownstream200 --mode gene`. We also took 200 base pairs up- and downstream regions from the gene boundaries into the analysis. For more details please see Kofler and Schlötterer (2012).

Using Gowinda led to only one significantly enriched category for the BBGP and no significant enrichment for the CMH test ($FDR < 0.05$; top ranked categories in Table S1, Table S2 and Supplementary Data for the full tables: GO_Gowinda_CMH.txt, GO_Gowinda_BBGP.txt).

5.2 Gene Set Enrichment with Mann-Whitney U Test

For using Gowinda, we had to fix a threshold above which we consider a SNP as a possible candidate. Defining this threshold can be arbitrary, and changes in the threshold can result in different enriched gene sets. Therefore, we decided to compare the distribution of all SNP-values in a specific gene set to the distribution outside that gene set using Mann-Whitney U test (MWU). This test allows us to decide if a particular gene set is significantly enriched based only on the ranks of SNP-values in that set.

We performed the MWU test similarly as Segrè *et al.* (2010). We used the previously mentioned gene set file obtained from R Bioconductor GO.db package; and a list of all SNPs with the corresponding values (output of the tests). For mapping the SNPs to the genes we used SNPEFF 2.0.1 (http://snpeff.sourceforge.net/). For each gene set we summarized the list of SNPs present in that particular set and created a vector of corresponding SNP-values (list of p-values or Bayes factors). Then we tested the alternative hypothesis that the distribution of these values is skewed towards the extreme values (low ranked p-values for the CMH, high ranked Bayes factors for the GP) compared to the values among the rest of the SNPs. This gives the observed rank-sum p-value for the investigated gene set. Then, similarly to Gowinda, we performed permutations to account for biases by simulating random gene sets (but keeping the chromosomal order) with identical size as observed. For every round of simulation, we calculated the ranked-sum p-values as before. Finally, an expected rank-sum p-value was computed from this null distribution, as the fraction of randomly sampled gene sets whose rank-sum p-value was less than or equal to the observed rank-sum p-value of the gene set.

The top ranked significant enrichments calculated with MWU test using 1000 permutations are functionally rather similar. Figure S19 shows the overlap between highly enriched categories for different empirical p-value cutoffs. The categories are listed in Table S3 and Table S4 and the full tables can be found in Supplementary Data: GO_MWU_CMH.txt and GO_MWU_BBGP.txt.

References

Agresti, A. (2002). *Categorical Data Analysis*. Wiley, New York.

Ashburner, M et al. (2000). Gene ontology: tool for the unification of biology. the gene ontology consortium. *Nat Genet*, **25**(1), 25–29.

Friendly, M. (2014). *vcdExtra: vcd extensions and additions*. R package version 0.6-0.

Kimura, M. (1962). On the probability of fixation of mutant genes in a population. *Genetics*, **47**, 713–719.

Kofler, R. and Schlötterer, C. (2012). Gowinda: unbiased analysis of gene set enrichment for genome-wide association studies. *Bioinformatics*, **28**, 2084–2085.

Kofler, R. and Schlötterer, C. (2014). A guide for the design of evolve and resequencing studies. *Mol Biol Evol*, **31**(2), 474–483.

Kuritz, SJ et al. (1988). A general overview of mantel-haenszel methods: applications and recent developments. *Annu Rev Public Health*, **9**, 123–160.

Orozco-Ter Wengel, P et al. (2012). Adaptation of Drosophila to a novel laboratory environment reveals temporally heterogeneous trajectories of selected alleles. *Molecular Ecology*, **21**, 4931–4941.

Segrè, A et al. (2010). Common inherited variation in mitochondrial genes is not enriched for associations with type 2 diabetes or related glycemic traits. *PLoS Genet.*, **6**, e1001058. doi:10.1371/journal.pgen.1001058.

6 Supplementary Tables and Figures

GO category	p-Value	FDR	Description
GO:0004003	0.000074	0.0630949	ATP-dependent DNA helicase activity
GO:0008094	0.0001048	0.0630949	DNA-dependent ATPase activity
GO:0006281	0.0002248	0.097873567	DNA repair
GO:0046914	0.000305	0.1027073	transition metal ion binding

Table S1: *Top ranked GO enrichment results with Gowinda on the CMH candidates.* Only the top 4 categories are shown. The full table is available as the Supplementary Data: GO_Gowinda_CMH.txt.

GO category	p-Value	FDR	Description
GO:0005506	0.0000143	0.015987	iron ion binding
GO:0015671	0.0004199	0.256548725	oxygen transport
GO:0004252	0.0006096	0.256548725	serine-type endopeptidase activity
GO:0004989	0.0007332	0.256548725	octopamine receptor activity

Table S2: *Top ranked GO enrichment results with Gowinda on the BBGP candidates.* Only the top 4 categories are shown. The full table is available as the Supplementary Data: GO_Gowinda_BBGP.txt.

GO category	Obs. p-val.	Emp. p-val.	Description
GO:0007274	2.8543e-156	0.001	neuromuscular synaptic transmission
GO:0032504	3.2726e-49	0.001	multicellular organism reproduction
GO:0006997	1.2159e-17	0.001	nucleus organization
GO:0007379	4.9304e-75	0.008	segment specification
GO:0003774	1.8303e-19	0.011	motor activity
GO:0009792	5.8937e-30	0.013	embryo development ending in birth or egg hatching
GO:0001700	9.7049e-31	0.015	embryonic development via the syncytial blastoderm
GO:0045451	4.5162e-20	0.015	pole plasm oskar mRNA localization
GO:0060810	2.3554e-19	0.015	intracell. mRNA loc. inv. in pattern specification proc.
GO:0060811	1.9679e-19	0.016	intracell. mRNA loc. inv. in anterior/posterior axis spec.
GO:0000975	1.5011e-32	0.017	regulatory region DNA binding
GO:0008298	5.7685e-17	0.017	intracellular mRNA localization
GO:0016573	3.4293e-08	0.024	histone acetylation
GO:0019094	6.8648e-19	0.025	pole plasm mRNA localization
GO:0060438	9.6931e-101	0.026	trachea development
GO:0000086	1.0455e-15	0.027	G2/M transition of mitotic cell cycle
GO:0030554	9.0394e-19	0.028	adenyl nucleotide binding
GO:0051049	4.8523e-52	0.029	regulation of transport
GO:0004386	1.9648e-09	0.029	helicase activity
GO:0007093	6.4409e-08	0.029	mitotic cell cycle checkpoint
GO:0032879	3.4419e-34	0.03	regulation of localization
GO:0060439	6.0698e-78	0.032	trachea morphogenesis
GO:0019904	3.6125e-74	0.032	protein domain specific binding
GO:0007350	1.1101e-25	0.033	blastoderm segmentation
GO:0000976	3.9652e-14	0.035	transcr.regulatory reg. sequence-spec. DNA binding
GO:0000977	2.8459e-28	0.037	RNA polymerase II reg. reg.seq.-spec. DNA binding
GO:0007276	3.7400e-24	0.038	gamete generation
GO:0007269	1.1198e-94	0.04	neurotransmitter secretion
GO:0004888	2.9136e-19	0.043	transmembrane signaling receptor activity
GO:0000981	1.9244e-28	0.044	seq.-spec DNA binding RNA pol. II transcr. factor activity
GO:0008306	1.2419e-35	0.046	associative learning
GO:0008355	6.2395e-32	0.047	olfactory learning
GO:0001012	1.3174e-37	0.048	RNA polymerase II regulatory region DNA binding
GO:0048149	1.6131e-23	0.048	behavioral response to ethanol
GO:0045664	7.9648e-23	0.048	regulation of neuron differentiation
GO:0010389	1.8391e-08	0.05	regulation of G2/M transition of mitotic cell cycle
GO:0009055	7.5572e-05	0.05	electron carrier activity

Table S3: *Results of the GO enrichment with MWU on the CMH candidates.* Only the categories are shown for which the empirical p-value ≤ 0.05 calculated for 1000 permutations. The full table is available as the Supplementary Data (GO_MWU_CMH.txt).

GO category	Obs. p-val.	Emp. p-val.	Description
GO:0006997	4.1404e-19	0	nucleus organization
GO:0007274	1.0657e-130	0.002	neuromuscular synaptic transmission
GO:0007379	3.7449e-85	0.002	segment specification
GO:0032879	8.0269e-38	0.006	regulation of localization
GO:0000075	1.9450e-19	0.007	cell cycle checkpoint
GO:0000785	9.1310e-15	0.014	chromatin
GO:0051049	6.3596e-52	0.019	regulation of transport
GO:0009152	2.7329e-41	0.02	purine ribonucleotide biosynthetic process
GO:0006164	5.9106e-46	0.022	purine nucleotide biosynthetic process
GO:0004386	1.0113e-09	0.025	helicase activity
GO:0005179	1.9714e-16	0.026	hormone activity
GO:0000975	2.2740e-25	0.027	regulatory region DNA binding
GO:0000977	9.8625e-36	0.028	RNA pol. II regulatory reg. seq.-spec. DNA binding
GO:0000976	2.6106e-18	0.029	transcr. reg. region sequence-spec.DNA binding
GO:0001012	2.0242e-42	0.029	RNA polymerase II regulatory region DNA binding
GO:0030554	1.9638e-14	0.03	adenyl nucleotide binding
GO:0046914	5.2243e-27	0.032	transition metal ion binding
GO:0055114	7.0135e-18	0.032	oxidation-reduction process
GO:0005829	1.0637e-17	0.033	cytosol
GO:0019725	2.3293e-26	0.034	cellular homeostasis
GO:0032504	8.4897e-21	0.036	multicellular organism reproduction
GO:0009165	8.8494e-25	0.038	nucleotide biosynthetic process
GO:0008285	7.1838e-19	0.041	negative regulation of cell proliferation
GO:0007269	1.6094e-94	0.043	neurotransmitter secretion
GO:0010389	3.4665e-07	0.043	regulation of G2/M transition of mitotic cell cycle
GO:0031226	5.5250e-27	0.043	intrinsic to plasma membrane
GO:0032940	3.7154e-73	0.045	secretion by cell
GO:0017076	5.6881e-12	0.046	purine nucleotide binding
GO:0000086	5.0627e-14	0.048	G2/M transition of mitotic cell cycle
GO:0016491	1.1667e-10	0.048	oxidoreductase activity

Table S4: *Results of the GO enrichment with MWU on the BBGP candidates.* Only the categories are shown for which the empirical p-value ≤ 0.05 calculated for 1000 permutations. The full table is available as the Supplementary Data (GO_MWU_BBGP.txt).

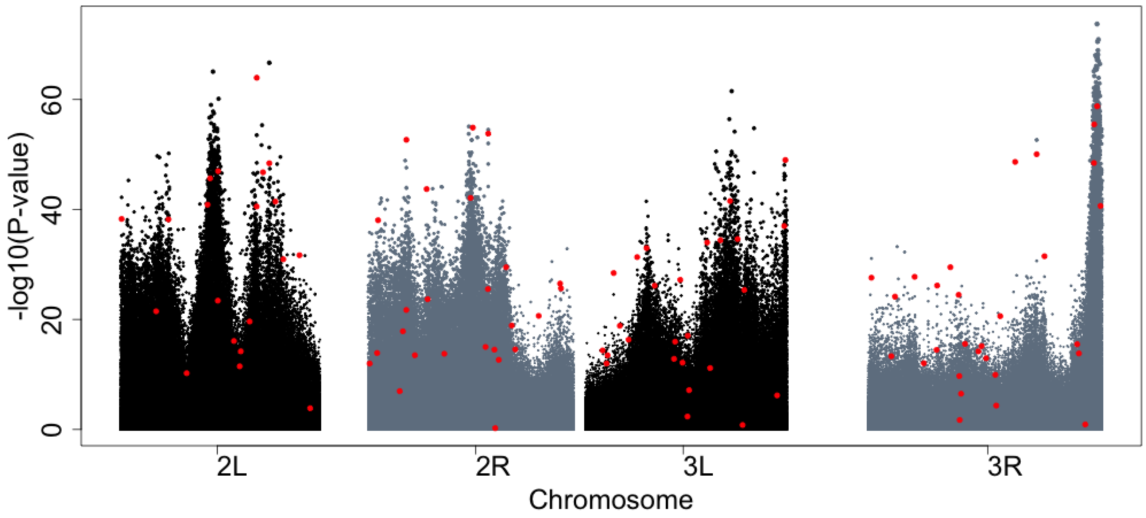

(a) Simulated data, genome-wide distribution of CMH -log(p-values).

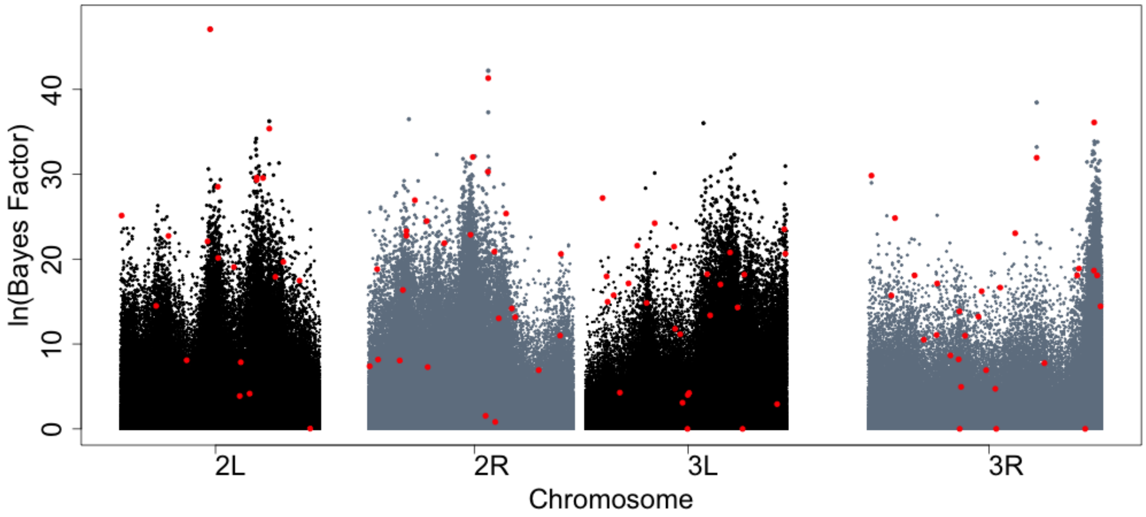

(b) Simulated data, genome-wide distribution of BBGP ln(Bayes factors).

Figure S1: *Manhattan plots of genome-wide test statistic values on simulated data with 5 replicates.* (a) -log(p-values) for the CMH test B-E comparison. (b) ln(Bayes factors) for the BBGP using 6 time points. Only autosomal regions were simulated and low recombining regions ($< 1cM/Mb$) were excluded. The 100 truly selected SNPs (s=0.1) are indicated in red. As the consequence of linkage structure, we observe extended peaks in the vicinity of selected SNPs. However, there are still some truly selected SNPs that do not show clear pattern of frequency increase. A possible explanation for that can be that the time course, i.e. 60 generations, is not long enough for them to rise significantly in frequency. They can also interfere with each other and non-selected SNPs.

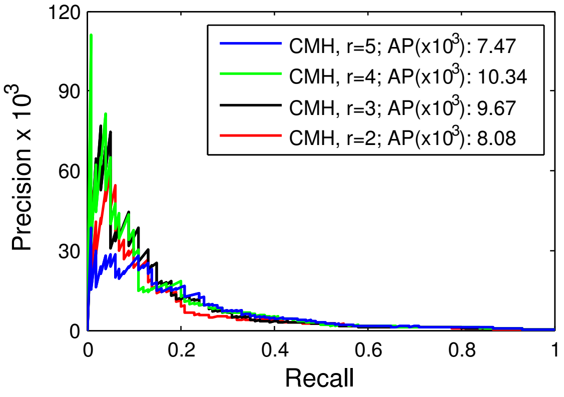

(a) CMH

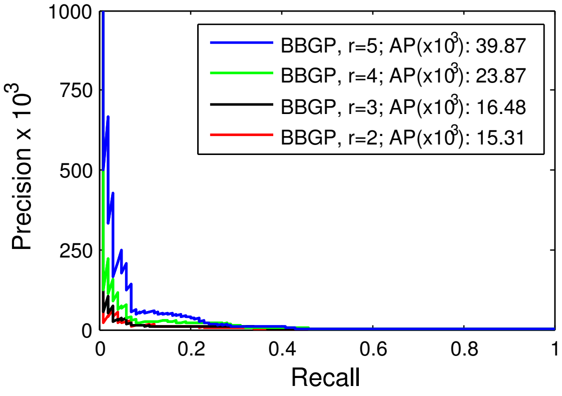

(b) BBGP (3 time points)

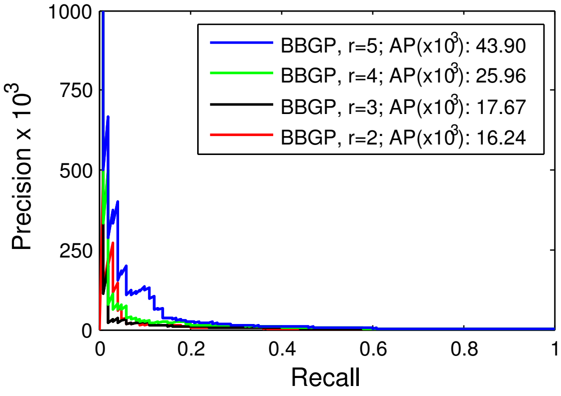

(c) BBGP (6 time points)

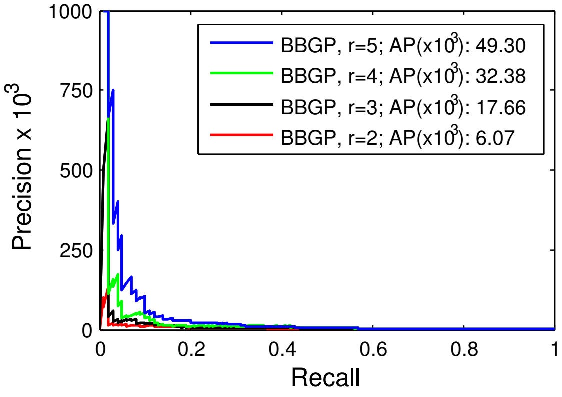

(d) BBGP (9 time points)

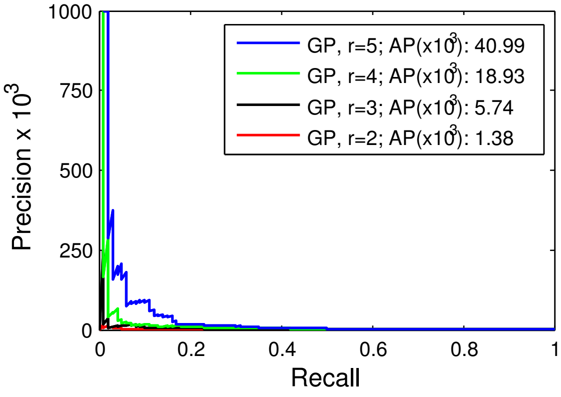

(e) GP (6 time points)

Figure S2: *Full precision-recall curves for the CMH and BBGP methods for Main Text Fig.5.* The precision is plotted as the function of recall for every possible cutoff value in the ranked sequence of candidate SNPs. The graph in Main Text Fig. 5 shows the average precisions for all replicate, time-point combinations.

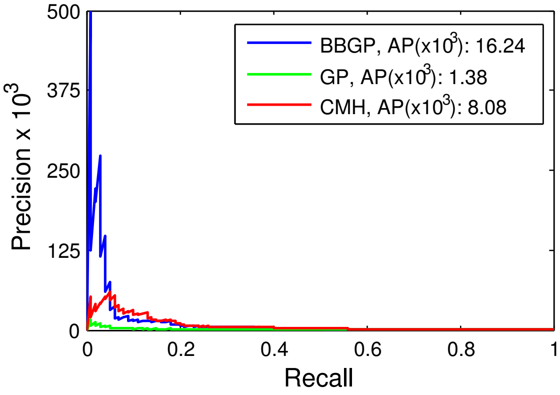

(a) Number of replicates: 2

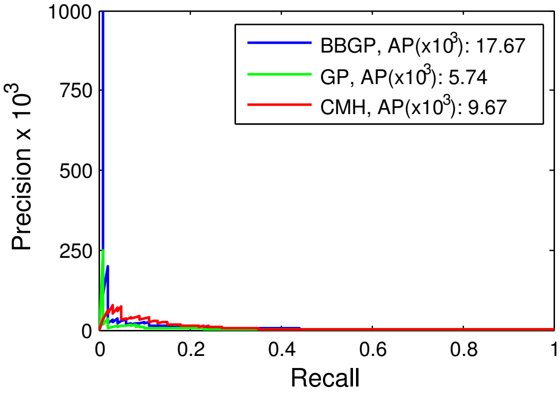

(b) Number of replicates: 3

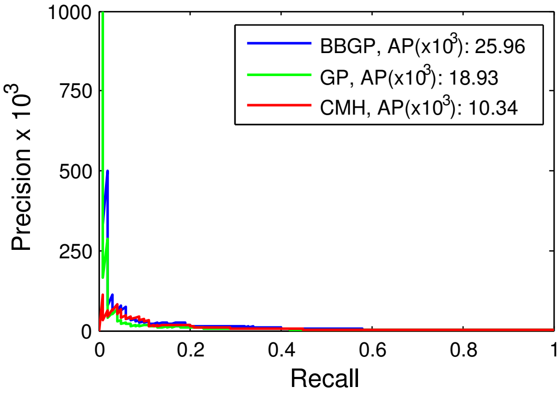

(c) Number of replicates: 4

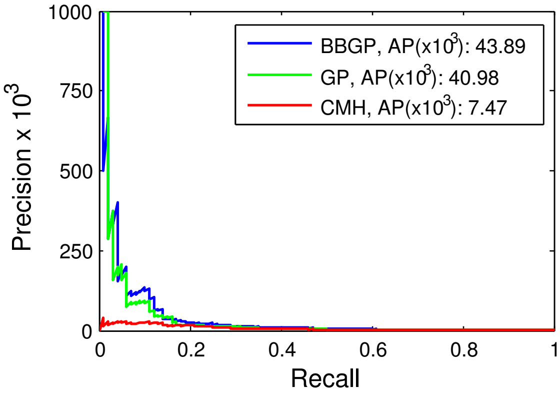

(d) Number of replicates: 5

Figure S3: *Precision recall curves comparing CMH method to the standard GP and BBGP methods using different number of replicates and 6 time points on whole-genome simulation.* Incorporation of the beta-binomial posterior variances into the GP model provides the most benefit when the number of replicates are small. The more replication is performed during the experiments, the better performance can be expected from the GP-based methods. The CMH test, however, does not benefit from more replicates in the same way.

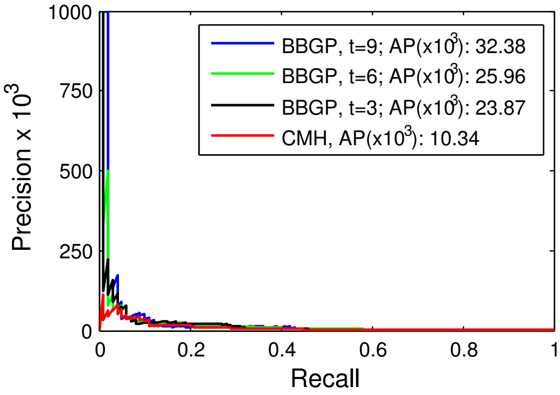

(a) Experiment: 1

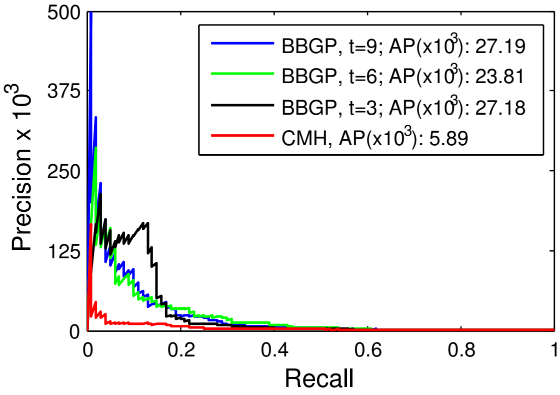

(b) Experiment: 2

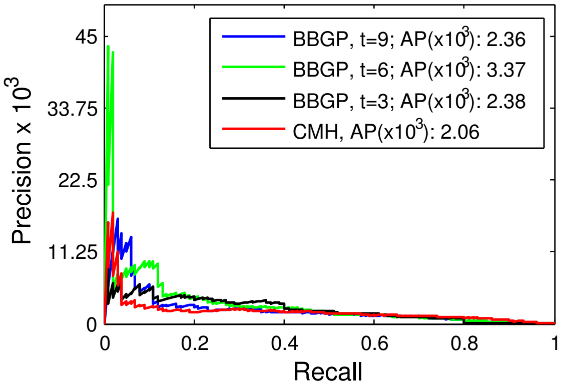

(c) Experiment: 3

Figure S4: *Precision recall curves comparing CMH to BBGP for 3 independent whole-genome experiments.* The performance can vary noticeably between experiments (e.g., factor of 10 difference in AP between Experiment 1 and 3). Nevertheless, the BBGP based test consistently outperforms the CMH test.

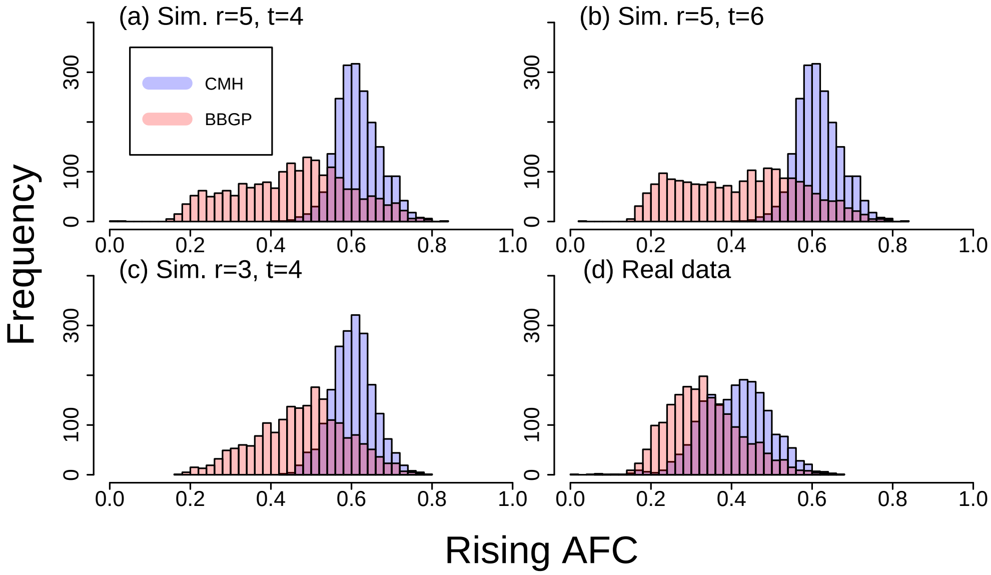

Figure S5: *Distribution of the average allele frequency change (AFC) of the rising allele for the top 2000 candidates in the whole-genome experiment.* AFC was calculated for each SNPs based on the average difference between the base and end populations across replicates. (a-b) AFC of the top 2000 candidates of the simulated data with 5 replicates, BBGP is performed on 4 (a) and 6 (b) time points, respectively. (c) AFC of the top 2000 candidates of the simulated data with 3 replicates, BBGP is performed on 4 time points. (d) AFC of the top 2000 candidates of the real data. We observed a significant location shift between the AFC distributions among the top 2000 candidate SNPs of the CMH and the BBGP (Mann-Whitney U, p-value $<$ 2.2e-16 for all panels). The location shift indicates that the CMH test mostly captures radical AFC while the GP-based methods are also sensitive to consistent signals coming from intermediate time points.

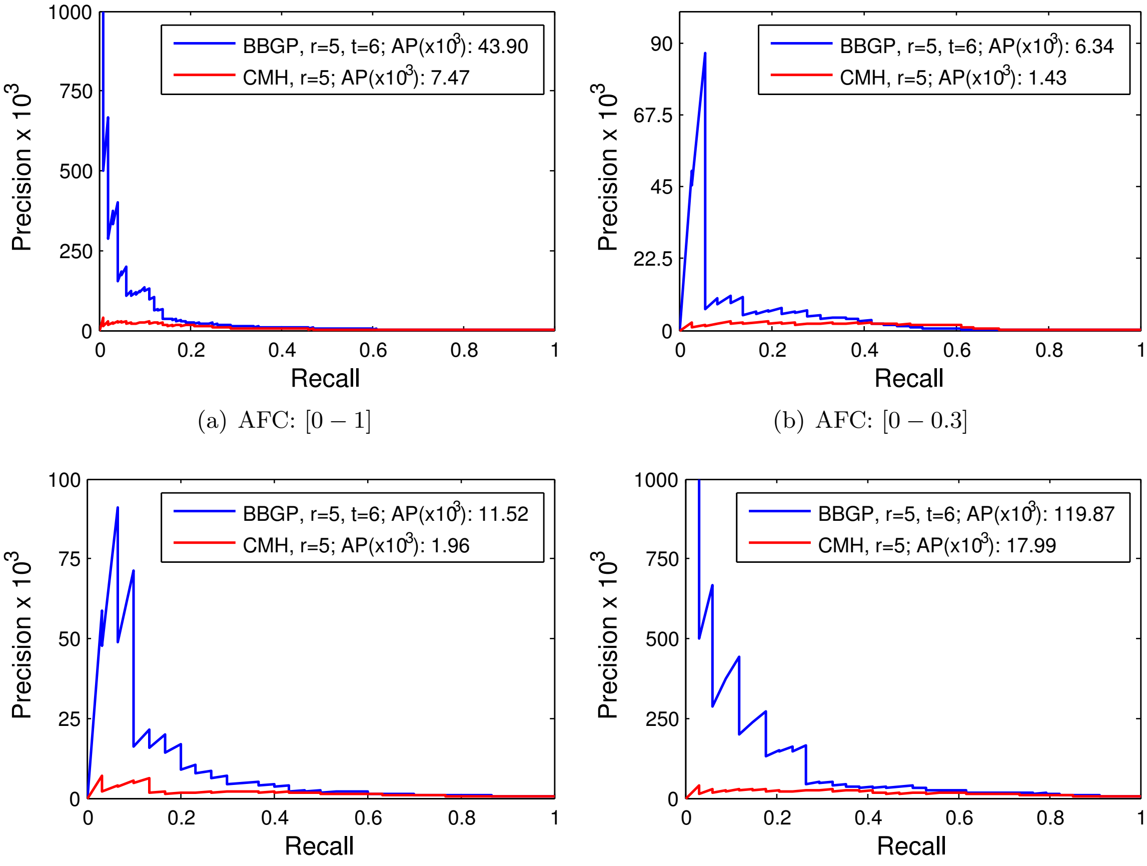

(a) AFC: $[0 - 1]$

(b) AFC: $[0 - 0.3]$

(c) AFC: $(0.3 - 0.5]$

(d) AFC: $(0.5 - 1]$

Figure S6: *Precision recall curves for different AFC classes in the whole-genome simulation.* The performance in terms of precision and recall is shown for the CMH and the BBGP in classes of SNPs with different allele frequency change. The AFC is measured between the base and end generations (60) and averaged over 5 replicates. 6 time points were used for the BBGP. Panel (a) shows the overall performance. In panels (b)-(d), the AFC classes contain the following number of selected SNPs: 36 in class [0-0.3], 30 in class (0.3-0.5], 34 in class (0.5-1.0].

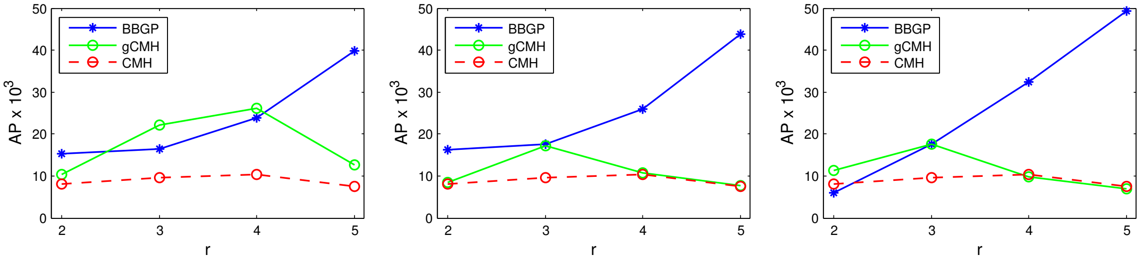

(a) t=3 (b) t=6 (c) t=9

Figure S7: *Average precision of the different methods with different number of time points (t) and replicates (r) in the whole-genome simulation.* Average precisions for the BBGP and the CMH test are same as on Main Text Fig. 5. Precisions of the generalised CMH test (gCMH) are added in green for every possible time-replicate combinations to the figures.

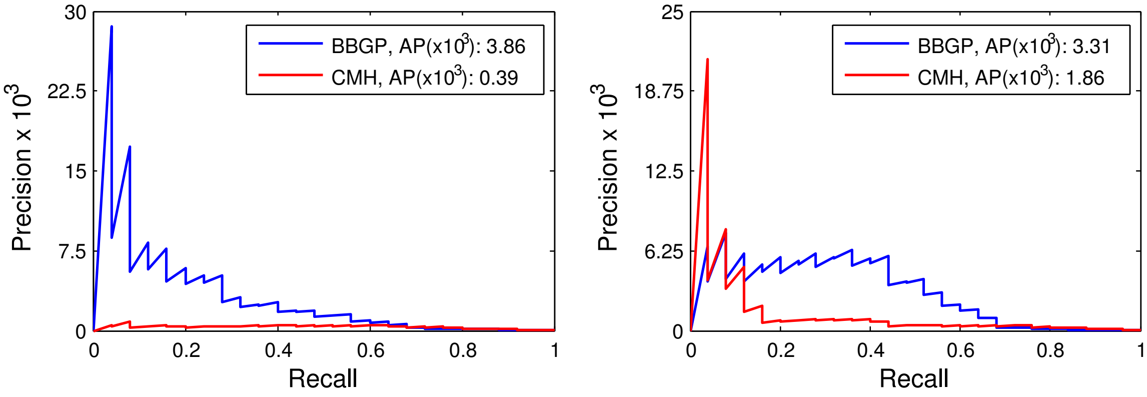

(a) H/N=0.5 (b) H/N=1

Figure S8: *Precision recall curves comparing CMH to BBGP for different H/N ratios for N=200 in the single-chromosome-arm simulation.*

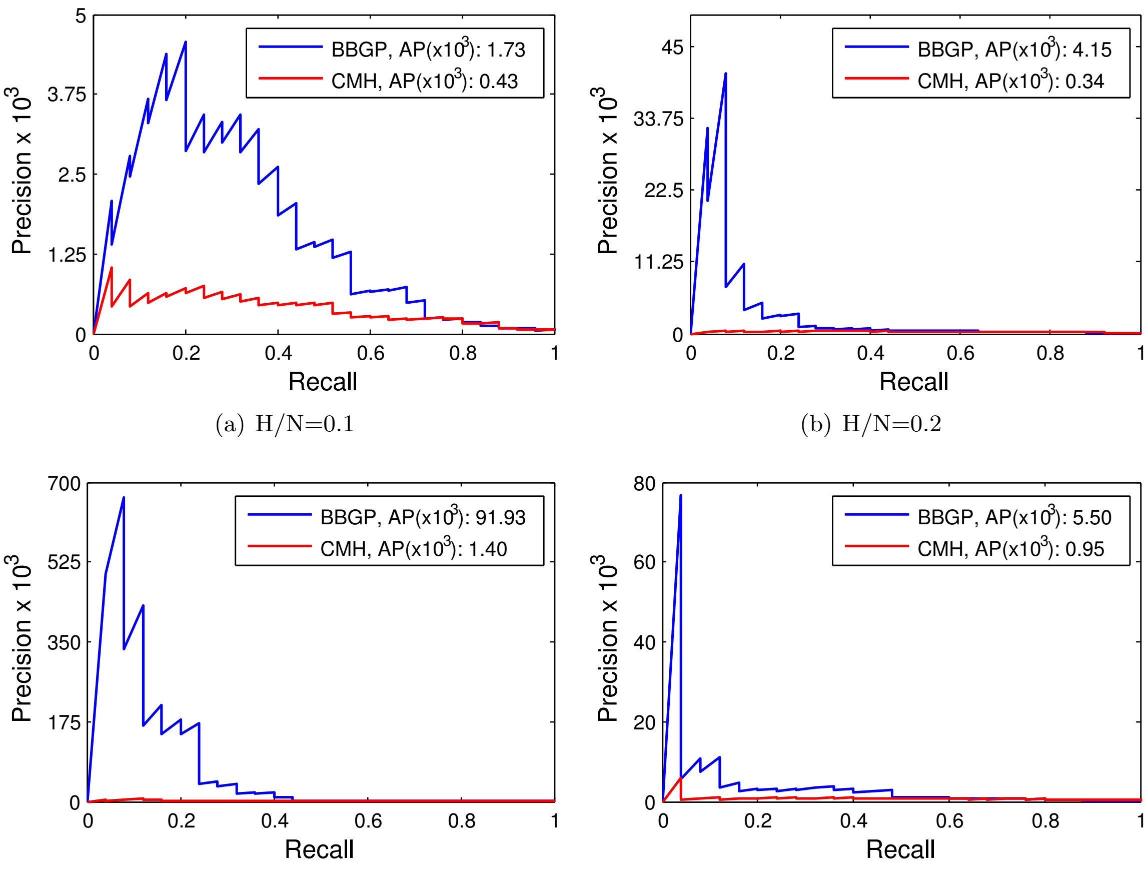

(a) H/N=0.1

(b) H/N=0.2

(c) H/N=0.5

(d) H/N=1

Figure S9: *Precision recall curves comparing CMH to BBGP for different H/N ratios for N=1000 in the single-chromosome-arm simulation.*

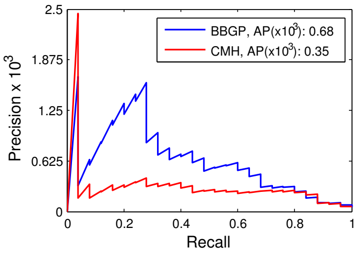

(a) H/N=0.02

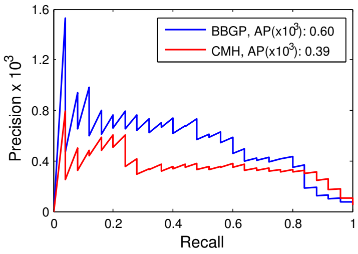

(b) H/N=0.04

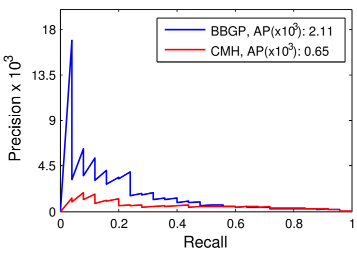

(c) H/N=0.1

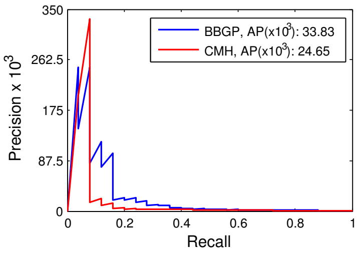

(d) H/N=0.2

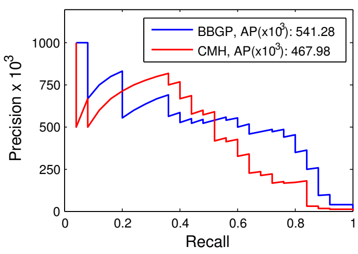

(e) H/N=0.5

Figure S10: *Precision recall curves comparing CMH to BBGP for different H/N ratios for N=5000 in the single-chromosome-arm simulation.*

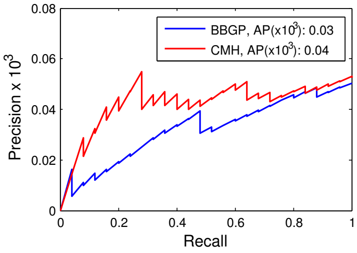

(a) s=0.005

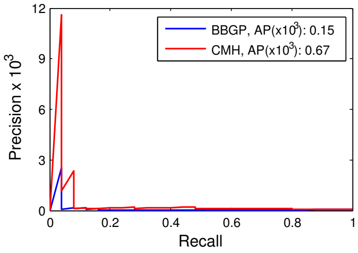

(b) s=0.01

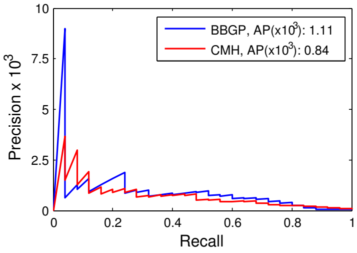

(c) s=0.05

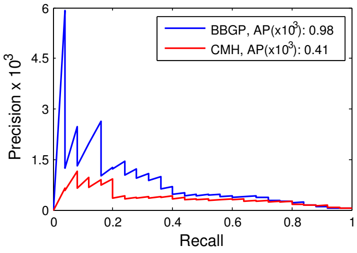

(d) s=0.07

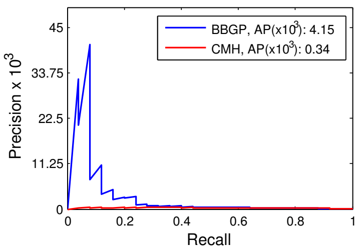

(e) s=0.1

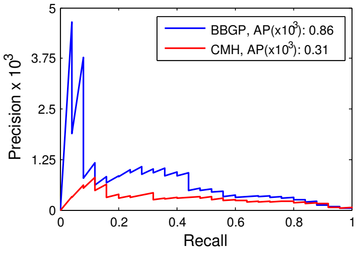

(f) s=0.14

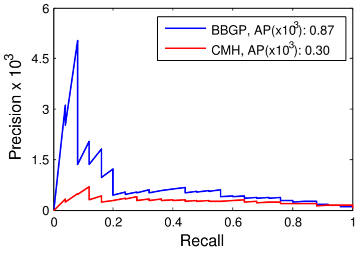

(g) s=0.2

Figure S11: *Precision recall curves comparing CMH to BBGP for different selection coefficients (s) in the single-chromosome-arm simulation.*

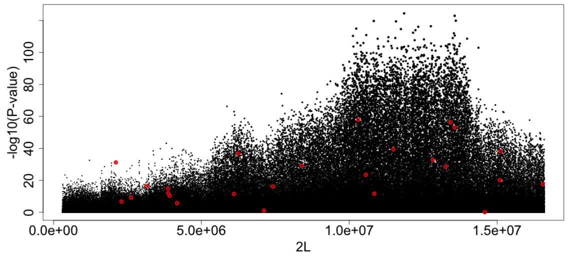

(a) $s = 0.1$, CMH -log(p-values) on 2L.

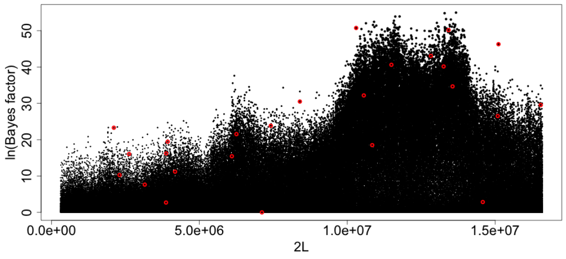

(b) $s = 0.1$, BBGP ln(Bayes factors) on 2L.

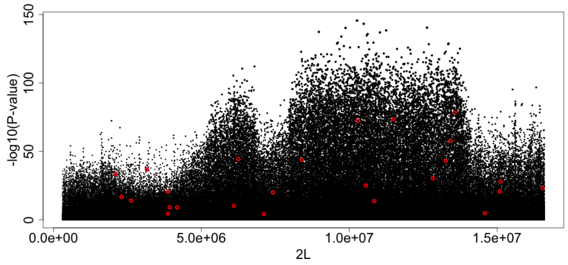

(c) $s = 0.2$, CMH -log(p-values).

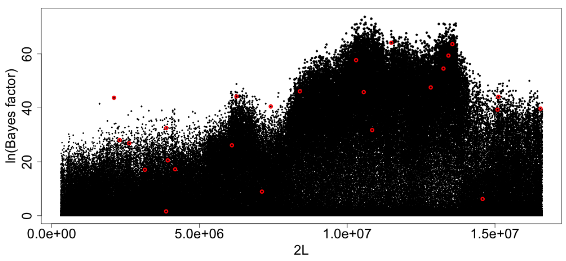

(d) $s = 0.2$, BBGP ln(Bayes factors) on 2L.

Figure S12: *Manhattan plots of test statistic values for simulations with a single chromosome arm.* (a,c) -log(p-values) for the CMH test B-G60 comparison for 5 replicates. (b,d) ln(Bayes factors) for the BBGP using 6 time points and 5 replicates. Truly selected SNPs (s=0.1 (a-b); s=0.2 (c-d)) are indicated in red.

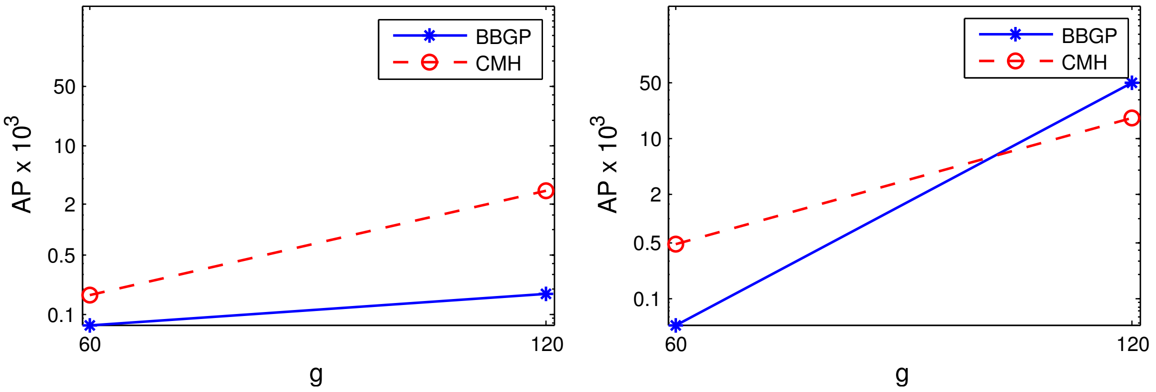

(a) s=0.005 (b) s=0.01

Figure S13: *Average precision with weak selection and large population size (N = 5000, H = 2500).* Log scale was used on y axis. The performance of the methods is shown when large populations evolved under weak selection. Under the basic parameter setup (Main Text Fig. 6 (b)) the CMH outperforms the BBGP for weak selection strength of $s = 0.005$ and 0.01. We observe the same behaviour even with larger population size ($N = 5000$, $H = 2500$) when the performance is evaluated using data up to generation 60. However, if we let the populations evolve further until generation 120, the BBGP gain a large performance improvement over the CMH test for $s = 0.01$. For weaker selection, we suppose that the BBGP would need even more time to outperform the CMH test.

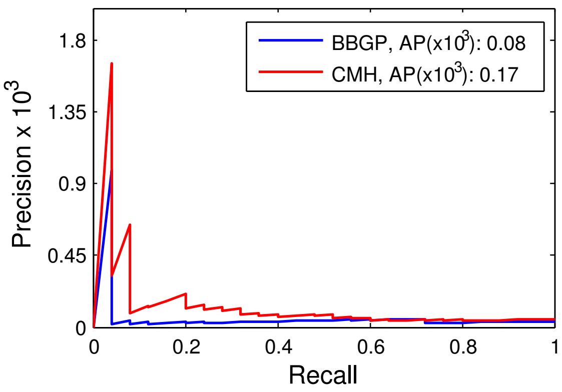

(a) s=0.005, g=60

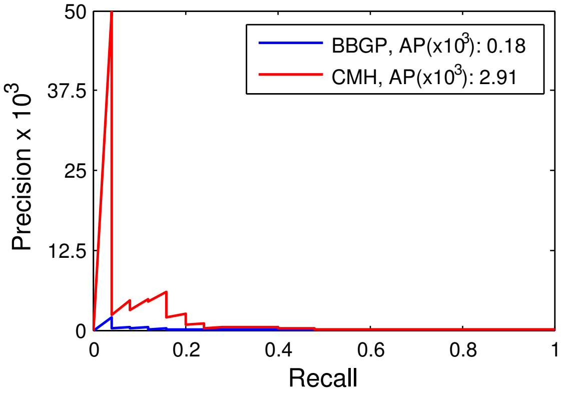

(b) s=0.005, g=120

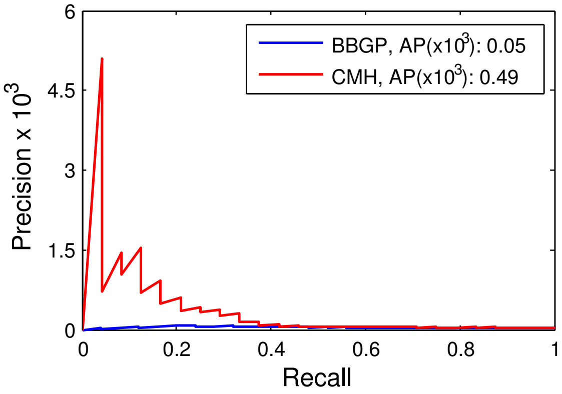

(c) s=0.01, g=60

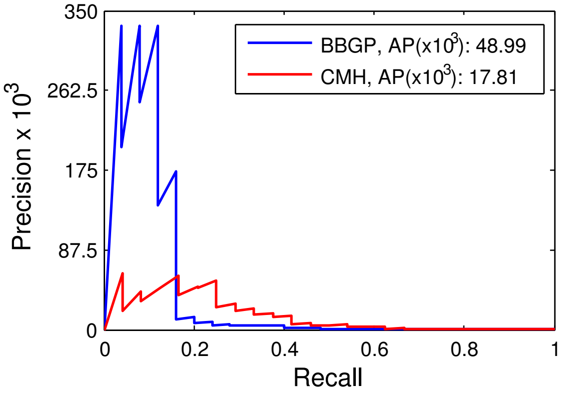

(d) s=0.01, g=120

Figure S14: *Precision recall curves comparing CMH to BBGP with weak selection for different time durations in the single-chromosome-arm simulation.* 6 time points were used in the BBGP: {0, 12, 24, 36, 48, 60} and {0, 24, 48, 72, 96, 120} for 60-generation and 120-generation experiments, respectively.

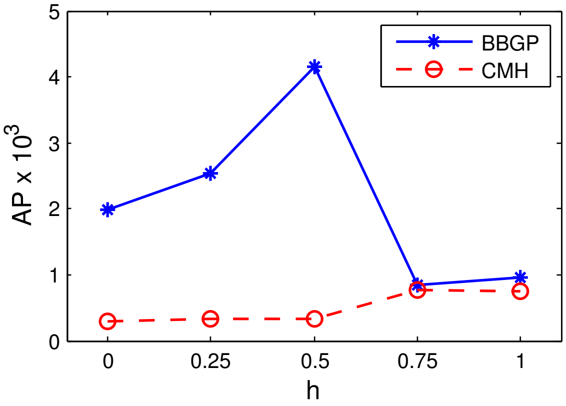

Figure S15: *Average precision for different levels of dominance (h) in the single-chromosome-arm simulation.*

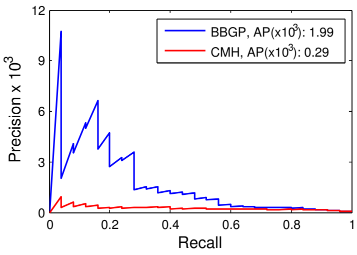

(a) h=0

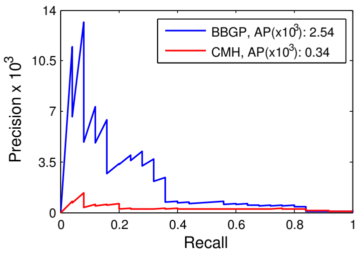

(b) h=0.25

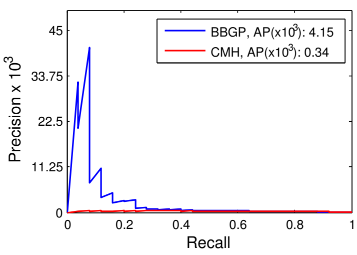

(c) h=0.5

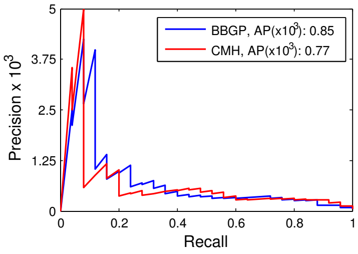

(d) h=0.75

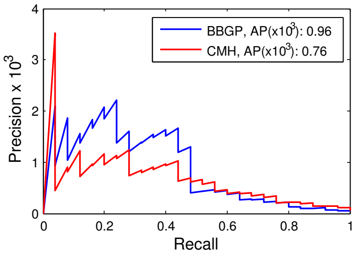

(e) h=1

Figure S16: *Precision recall curves comparing CMH to BBGP for different dominance levels (h) in the single-chromosome-arm simulation.*

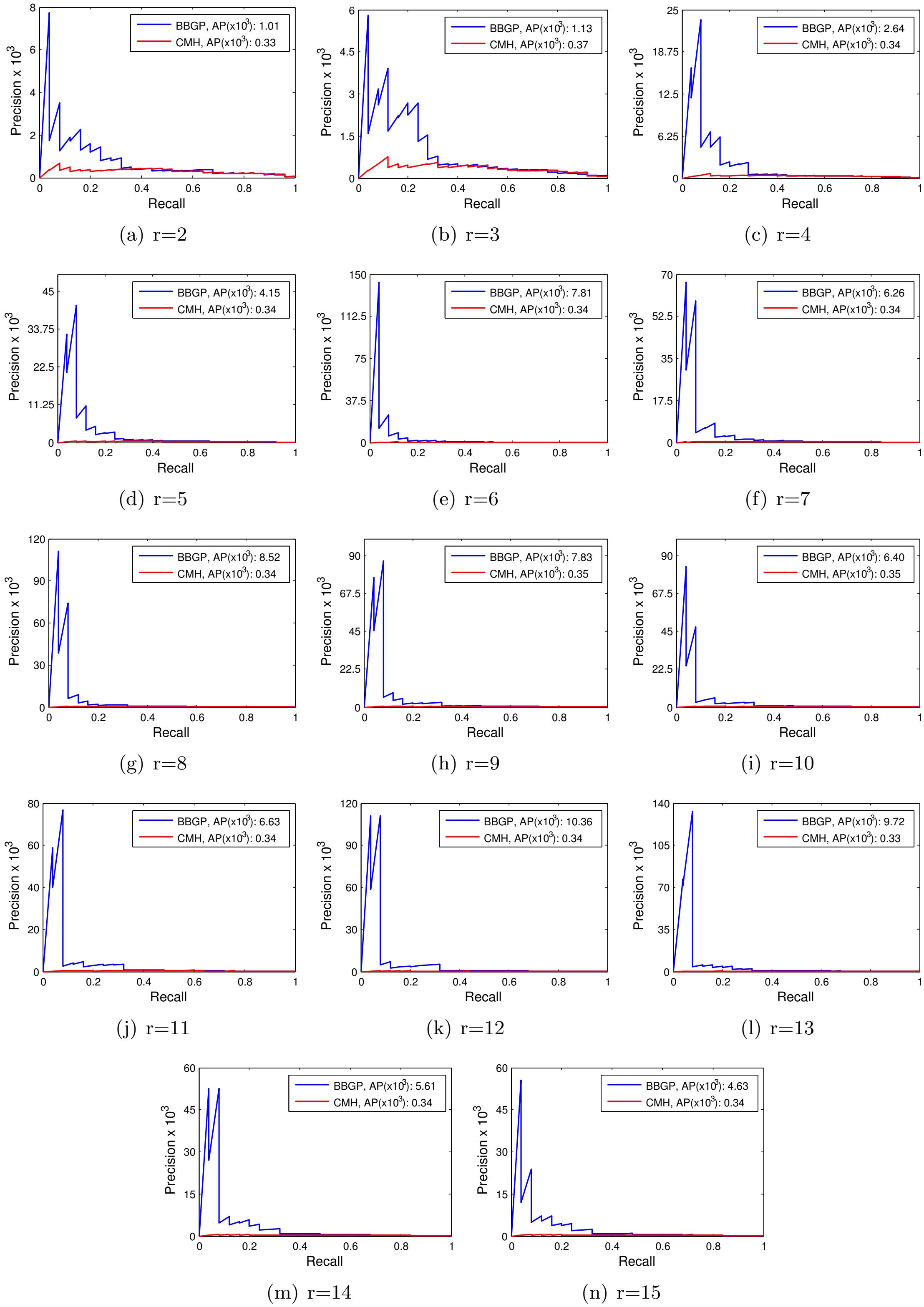

Figure S17: *Precision recall curves comparing CMH to BBGP for different number of replicates (r) in the single-chromosome-arm simulation.*

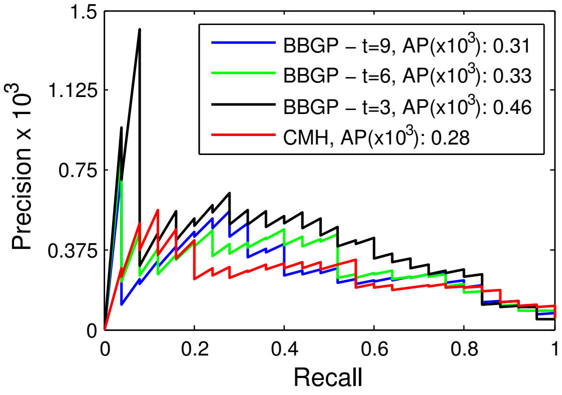

(a) G0-G30

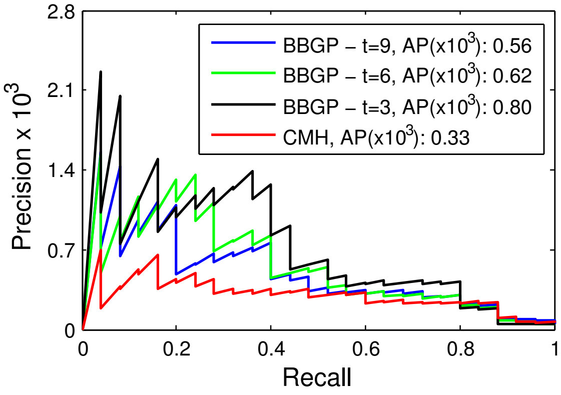

(b) G0-G60

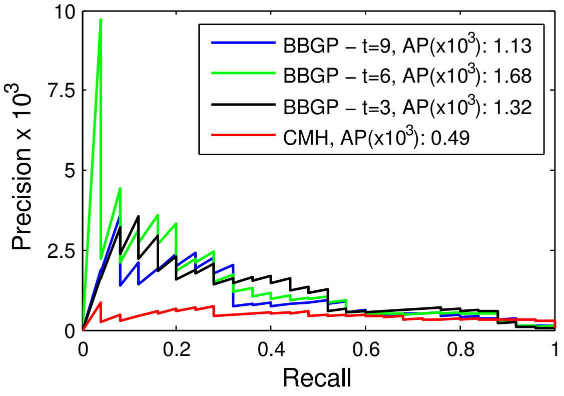

(c) G0-G120

Figure S18: *Precision recall curves comparing CMH to BBGP using different number of time points combined with different experiment lengths (single-chromosome-arm simulation).* In order to investigate the effects of the time spacing as well as the duration of the experiment, the following sampling schemes were applied on the time points:
G0-G30: {0, 18, 30}, {0, 6, 12, 18, 24, 30}, {0, 4, 6, 10, 14, 18, 22, 26, 30} ;
G0-G60: {0, 36, 60}, {0, 12, 24, 36, 48, 60}, {0, 8, 12, 20, 28, 36, 44, 52, 60} ;
G0-G120: {0, 72, 120}, {0, 24, 48, 72, 96, 120}, {0, 16, 24, 40, 56, 72, 88, 104, 120}.

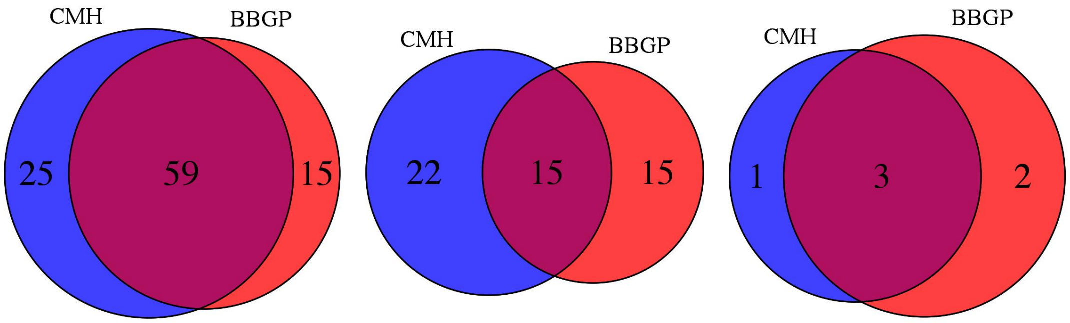

(a) Emp. p-value ≤ 0.1 (b) Emp. p-value ≤ 0.05 (c) Emp. p-value ≤ 0.01

Figure S19: *Venn diagram of significantly enriched GO categories.* Empirical p-values (Emp. p-val.) for the MWU tests are calculated for each category based on sampling random SNPs (1000 times) but keeping their chromosomal order. Overlaps between CMH and BBGP tests are shown for different significance levels.

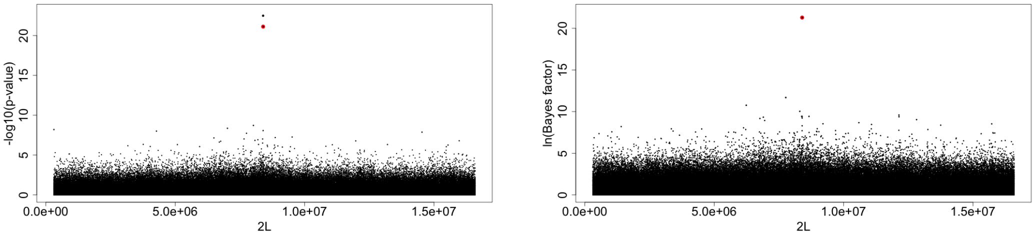

(a) CMH test (b) BBGP

Figure S20: *Manhattan plots on simulated data using only a single selected SNP ($s = 0.1$) on the whole chromosome arm.* Simulation was performed as described in Main Text Section 2.7 on a single chromosome arm of 2L ($\sim 16Mb$) using the basic parameter setup. The only difference is the number of SNPs assigned to be selected. Here we used a single selected SNP on the middle of the chromosome (highlighted in red) to see how much influence does the interference between selected SNPs play in shaping the dynamics of allele frequency trajectories. We see striking evidence that high number of false positives are due to interactions between linked selected sites.

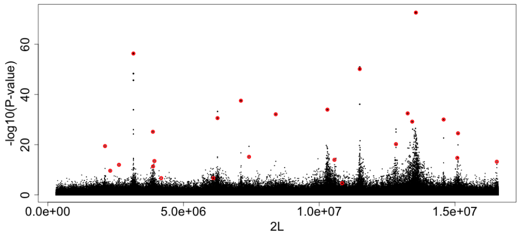

(a) CMH test

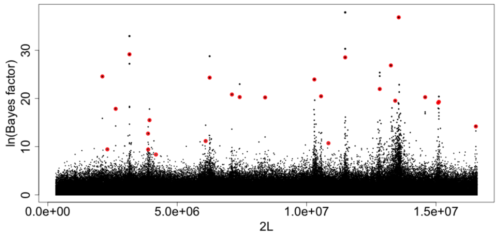

(b) BBGP

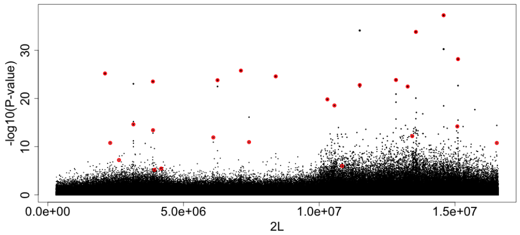

(c) CMH test

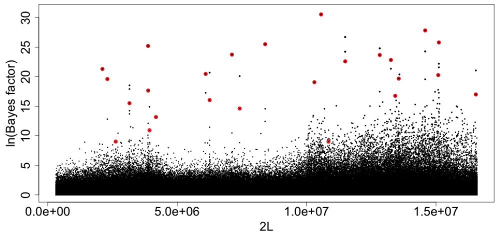

(d) BBGP

Figure S21: *Manhattan plots with high recombination rate (a-b) and large population size (c-d).* Top row (a-b): Simulation, as described in Main Text Section 2.7, was carried out by setting high recombination rate uniformly across 2L. Bottom row (c-d): Simulation with normal level of recombination but using large populations size of $N = 5000, H = 2500$. Selected SNPs are indicated in red. Linkage is broken up when large population size is used for simulations (c-d) and the dynamics of allele trajectories become more similar to the ones that are simulated with high recombination rate (a-b). For experimental design, however, recombination rates cannot be easily modified but similar effect can be attained by propagating larger populations.

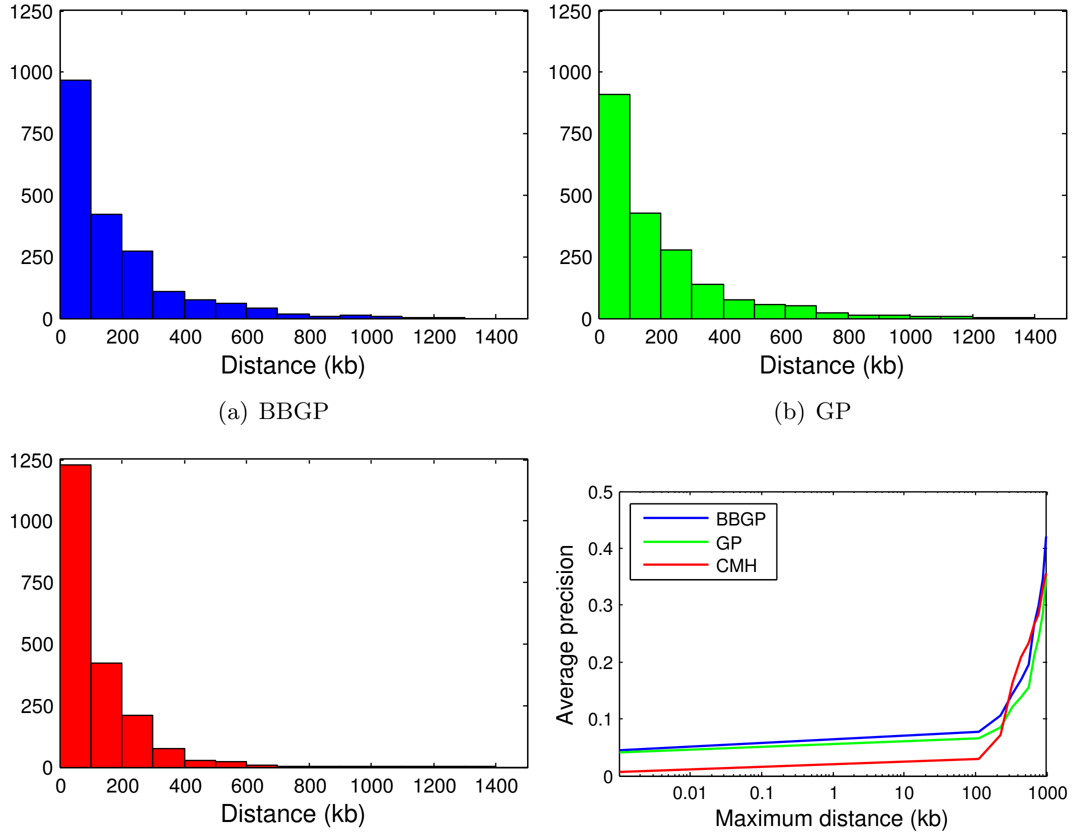

(a) BBGP (b) GP

(c) CMH (d) Average precisions

Figure S22: *Distribution of the distances (kb) to the nearest selected SNPs for the top 2000 candidate SNPs (a-c) and average precisions when potential hitchhikers are excluded (d).* The lines in panel (d) show the performances of the methods when the potential hitchhikers, i.e. non-selected SNPs closer than the given distance from a selected SNP, are excluded prior to the calculation of the average precisions. Log-scale was used on x-axis, which shows the maximum distance (kb) of the excluded potential hitchhikers to the nearest selected SNPs. The plots were obtained from whole-genome simulation data with 5 replicates and 6 time points.

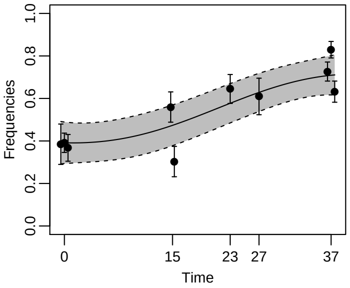

(a) BBGP rank: 86237
CMH rank: 1

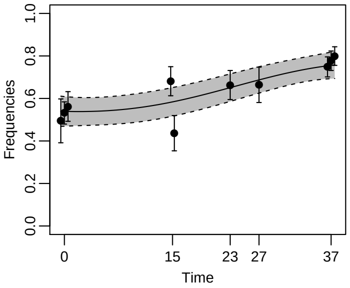

(b) BBGP rank: 104021
CMH rank: 43

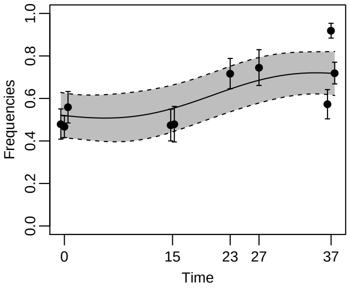

(c) BBGP rank: 315941
CMH rank: 49

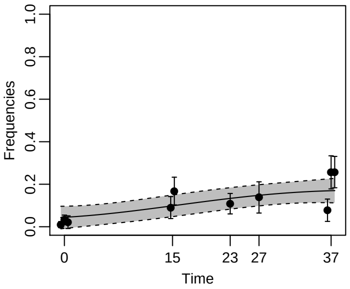

(d) BBGP rank: 271348
CMH rank: 60

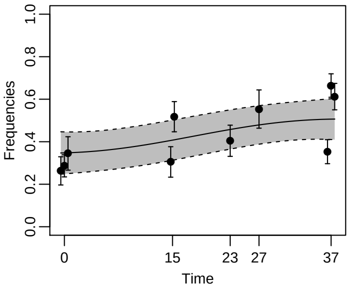

(e) BBGP rank: 442855
CMH rank: 61

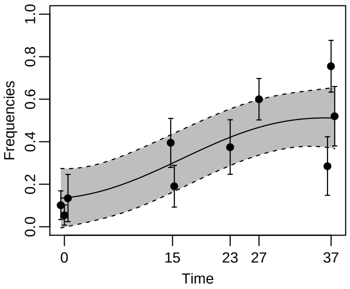

(f) BBGP rank: 172516
CMH rank: 85

Figure S23: *Candidate SNPs from the real data which were ranked in the top of the list by CMH but in the bottom of the list by BBGP.* Ranks in each method are shown for each example candidate. Confidence regions are shown for ± 2 standard deviation. Similarly, error bars indicate ± 2 standard deviation (from FBB) interval. Replicates at the same time points are shifted by 0.5 for better visualisation.

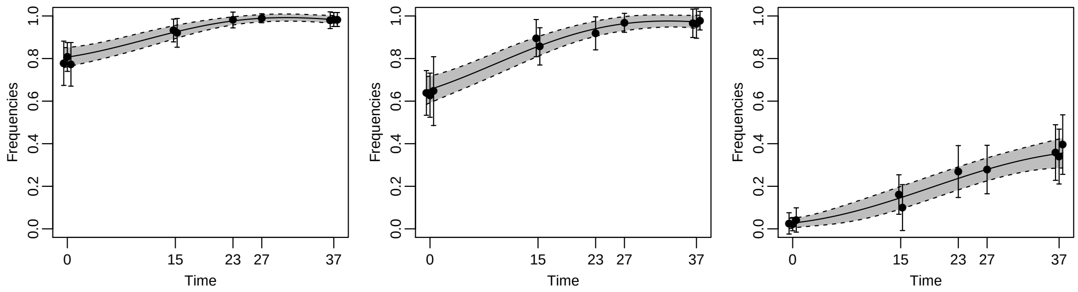

(a) BBGP rank: 60
CMH rank: 12982

(b) BBGP rank: 74
CMH rank: 7142

(c) BBGP rank: 99
CMH rank: 1322

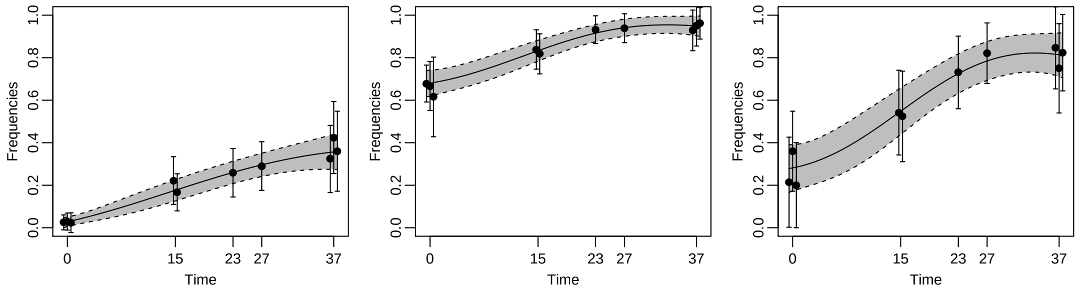

(d) BBGP rank: 130
CMH rank: 1319

(e) BBGP rank: 650
CMH rank: 37263

(f) BBGP rank: 1940
CMH rank: 24026

Figure S24: *Candidate SNPs from the real data which were ranked in the top of the list by BBGP but in the bottom of the list by CMH.* Ranks in each method are shown for each example candidate. Confidence regions are shown for ± 2 standard deviation. Similarly, error bars indicate ± 2 standard deviation (from FBB) interval. Replicates at the same time points are shifted by 0.5 for better visualisation.

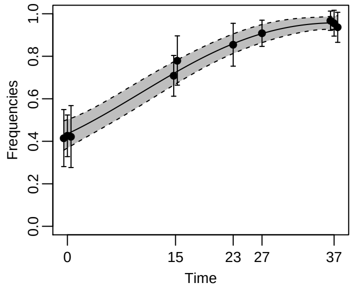

(a) BBGP rank: 1
CMH rank: 23

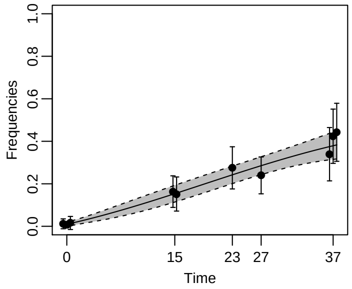

(b) BBGP rank: 2
CMH rank: 4

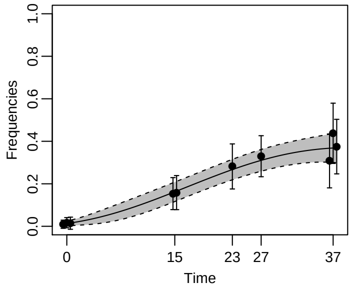

(c) BBGP rank: 3
CMH rank: 15

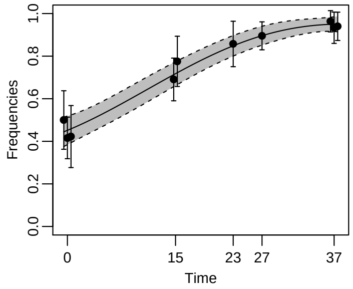

(d) BBGP rank: 7
CMH rank: 57

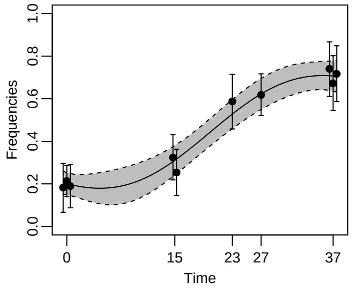

(e) BBGP rank: 39
CMH rank: 82

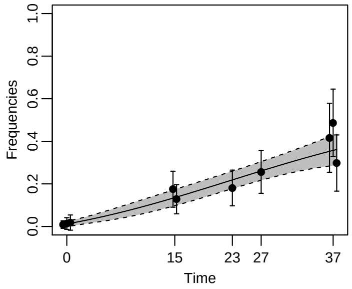

(f) BBGP rank: 43
CMH rank: 39

Figure S25: *Candidate SNPs from the real data which were ranked in the top of the list both by CMH and BBGP.* Ranks in each method are shown for each example candidate. Confidence regions are shown for ± 2 standard deviation. Similarly, error bars indicate ± 2 standard deviation (from FBB) interval. Replicates at the same time points are shifted by 0.5 for better visualisation.
